# Automated Discovery of Functional Generality of Human Gene Expression Programs

**DOI:** 10.1371/journal.pcbi.0030148

**Published:** 2007-08-10

**Authors:** Georg K Gerber, Robin D Dowell, Tommi S Jaakkola, David K Gifford

**Affiliations:** 1 Department of Computer Science and Electrical Engineering, Massachusetts Institute of Technology, Cambridge, Massachusetts, United States of America; 2 Harvard–Massachusetts Institute of Technology Division of Health Sciences and Technology, Cambridge, Massachusetts, United States of America; 3 Whitehead Institute for Biomedical Research, Cambridge Massachusetts, United States of America; Stanford University, United States of America

## Abstract

An important research problem in computational biology is the identification of *expression programs,* sets of co-expressed genes orchestrating normal or pathological processes, and the characterization of the functional breadth of these programs. The use of human expression data compendia for discovery of such programs presents several challenges including cellular inhomogeneity within samples, genetic and environmental variation across samples, uncertainty in the numbers of programs and sample populations, and temporal behavior. We developed GeneProgram, a new unsupervised computational framework based on Hierarchical Dirichlet Processes that addresses each of the above challenges. GeneProgram uses expression data to simultaneously organize tissues into groups and genes into overlapping programs with consistent temporal behavior, to produce maps of expression programs, which are sorted by generality scores that exploit the automatically learned groupings. Using synthetic and real gene expression data, we showed that GeneProgram outperformed several popular expression analysis methods. We applied GeneProgram to a compendium of 62 short time-series gene expression datasets exploring the responses of human cells to infectious agents and immune-modulating molecules. GeneProgram produced a map of 104 expression programs, a substantial number of which were significantly enriched for genes involved in key signaling pathways and/or bound by NF-κB transcription factors in genome-wide experiments. Further, GeneProgram discovered expression programs that appear to implicate surprising signaling pathways or receptor types in the response to infection, including Wnt signaling and neurotransmitter receptors. We believe the discovered map of expression programs involved in the response to infection will be useful for guiding future biological experiments; genes from programs with low generality scores might serve as new drug targets that exhibit minimal “cross-talk,” and genes from high generality programs may maintain common physiological responses that go awry in disease states. Further, our method is multipurpose, and can be applied readily to novel compendia of biological data.

## Author Summary

In recent years, DNA microarrays have been used to produce large compendia of human gene expression data, which are promising resources for discovery of *expression programs,* sets of co-expressed genes orchestrating important physiological or pathological processes. However, these compendia present particular challenges, including cellular inhomogeneity within samples, genetic and environmental variation across samples, uncertainty in the numbers of programs and sample populations, and temporal behavior. To address these challenges, we developed GeneProgram, a state-of-the-art statistical framework that automatically generates interpretable maps of expression programs from microarray data. GeneProgram accomplishes this by simultaneously organizing tissues into groups and genes into overlapping programs with consistent temporal behavior, and sorting programs by a generality score. Such maps may be valuable for guiding future biological experiments; genes from programs with low generality scores might serve as new drug targets that exhibit minimal “cross-talk,” and genes from high generality programs may maintain common physiological responses that go awry in disease states. Using synthetic and real data, we showed that GeneProgram outperformed several popular expression analysis methods. Further, on a compendium of time-series gene expression data measuring the responses of human cells to infectious agents, GeneProgram discovered programs that implicate surprising signaling pathways and receptor types.

## Introduction

The great complexity of the human body, in both normal physiology and pathological states, arises from the coordinated expression of genes. A fundamental challenge in computational biology is the identification of sets of co-activated genes in a given biological context and the characterization of the functional breadth of such sets. Understanding of the functional generality of gene sets has both practical and theoretical utility. Sets of genes that are very specific to a particular cell type or pathological condition may be useful as diagnostic markers or drug targets. In contrast, sets of genes that are active across diverse cell types or pathological states can give us insight into unexpected functional similarities and involvement of core common pathways.

In this study, we use a large compendium of short time-series gene expression datasets measuring the responses of human cells to infectious agents or immune-modulating molecules, to discover a set of biologically interpretable expression programs and to characterize quantitatively the specificity of each program. Such large genome-wide human expression data compendia present several new challenges that do not necessarily arise when analyzing data from simpler organisms. First, tissue samples may represent collections of diverse cell-types mixed together in different proportions. Even if a sample consists of a relatively homogenous cell population, the cells can still behave asynchronously. Second, each tissue sample is often from a different individual, so that the compendium represents a patchwork of samples from different genetic and environmental backgrounds. Third, the number of expression programs and distinct cell populations present in a compendium is effectively unknown a priori. Fourth, a compendium may contain experiments measuring temporal responses over different durations or using varied sampling rates.

We present a novel methodology, GeneProgram, designed for analyzing large compendia of human expression data, which simultaneously compresses sets of genes into expression programs and sets of tissues into groups. Specific features of our algorithm address each of the above issues relating to analysis of compendia of human gene expression data.

First, GeneProgram handles tissue inhomogeneity by allocating the total mRNA recovered from each tissue to different gene expression programs, which may be shared across tissues. The number of expression programs used by a tissue therefore relates to its functional homogeneity. Second, GeneProgram handles the disparate origin of tissue samples by explicitly modeling each tissue as a probabilistic sample from a population of related tissues. Related tissues are assumed to use similar expression programs and to similar extents, but the precise number of genes and the identity of genes used from each program may vary in each sample. Additionally, populations of related tissues are discovered automatically, and provide a natural means for characterizing the generality of expression programs. Third, GeneProgram handles uncertainty in the numbers of tissue groups and expression programs by using a non-parametric Bayesian technique, Dirichlet Processes, which provides prior distributions over numbers of sets. Fourth, GeneProgram explicitly models patterns of temporal expression change using the novel concept of program *usage modifiers*. A usage modifier is a variable that is specific to a tissue-expression program pair and describes how a tissue uses the program. For instance, usage modifiers can specify the temporal phase and direction (induction or repression) of expression. Thus, the genes used by a tissue from a program and the manner in which they are expressed (e.g., early induction versus late repression) are chosen probabilistically and influenced by the behavior of similar tissues. Further, usage is by definition consistent across a program for a particular tissue, which facilitates biological interpretation.

To understand the novelty of GeneProgram, it is useful to view our framework in the context of a lineage of unsupervised learning algorithms that have previously been applied to gene expression data. These algorithms are diverse, and can be classified according to various features, such as whether they use matrix factorization methods [1], heuristic scoring functions [2], generative probabilistic models [3], statistical tests [4,5], or some combinations of these methods [6,7]. The simplest methods, such as K-means clustering, assume that all genes in a cluster are co-expressed across all tissues, and that there is no overlap among clusters. Next in this lineage are biclustering algorithms [2,5,8–10], which assume that all genes in a bicluster are co-expressed across a subset rather than across all tissues. In many such algorithms, genes can naturally belong to multiple biclusters.

GeneProgram is based on two newer unsupervised learning frameworks, the *topic model* [11,12] and the Hierarchical Dirichlet Process mixture model [13]. The topic model formalism allows GeneProgram to further relax the assumptions of typical biclustering methods, through a probabilistic model in which each gene in an expression program has a (potentially) different chance of being co-expressed in a subset of tissues. The hierarchical structure of our model, which encodes the assumption that groups of tissues are more likely to use similar sets of expression programs in similar proportions, also provides advantages. Hierarchical models tend to be more robust to noise, because statistical strength is “borrowed” from items in the same group for estimating the parameters of clusters. Additionally, hierarchical models can often be interpreted more easily—in the context of the present application, the inferred expression programs will tend to be used by biologically coherent sets of tissues or experimental conditions. Finally, through the Dirichlet Process mixture model formalism, GeneProgram automatically infers the numbers of gene expression programs and tissue groups. Because this approach is fully Bayesian, the numbers of mixture components can be integrated over during model learning, so that the complexity of the model is appropriately penalized. This is in contrast to previous methods that either require the user to specify the number of clusters directly or produce as many clusters as are deemed significant with respect to a heuristic or statistical score without providing a global complexity penalty. We note that Medvedovic et al. have also applied Dirichlet Process mixture models to gene expression analysis, but not in the context of topic models, of Hierarchical Dirichlet Processes, or to human data [14].

A variety of algorithms have been developed to analyze time-series expression data [15], but, to our knowledge, none have been specifically designed for analysis of large compendia of such data. Analysis methods for combining time-series of different durations or that use different sampling rates have focused on long time-series over a few experimental conditions [16,17], rather than on short series over many conditions as we do. Jenner and Young performed a meta-analysis using hierarchical clustering of a superset of the infection time-course experiments we analyze in this paper [18]. However, their analysis was not automated or statistically principled, relying on extensive prior biological knowledge, and using visual assessment of clusters to manually assign genes to pathways of interest. Further, as described in the Results/Discussion section, our analysis implicated several surprising signaling pathways and receptor types in the response to infection that previous analyses of these datasets have not.

In the remainder of this paper, we describe the GeneProgram algorithm, and the biological insights gained from applications of our method. We first present an intuitive overview of the GeneProgram algorithm and probability model. We then use synthetic data to examine the kinds of structures that GeneProgram and several other classes of algorithms can recover from noisy data. Next, we benchmark GeneProgram on two large compendia of mammalian data [19,20] by comparing our algorithm's ability to recover biologically relevant gene sets to those of two popular biclustering methods. We then apply GeneProgram to a compendium of 62 short time-series gene expression datasets, measuring the responses of human cells to infectious agents or immune-modulating molecules [21–26], and produce a map of expression programs organized by functional generality scores. We evaluate the biological relevance of the discovered expression programs using biological process categories [27] and pathway [28] databases, as well as genome-wide data profiling binding of NF-κB family transcription factors [29]. Finally, we provide examples of discovered expression programs involved in key pathways related to the response to infection, discuss the significance of our results, and comment on possible future research directions.

## Results/Discussion

### The GeneProgram Algorithm and Probability Model

#### Algorithm overview.

**Figure 1:**
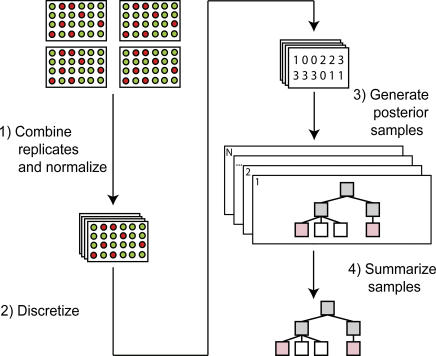
GeneProgram Algorithm Steps The main steps of the algorithm are: data pre-processing (steps 1–2), model inference/learning (step 3), and model posterior distribution summarization (step 4). See the text for details.

The GeneProgram algorithm consists of data pre-processing, model inference/learning, and model posterior distribution summary steps as depicted in Figure 1. Data pre-processing steps make data from multiple experiments comparable and discretize continuous values in preparation for input to the model. The model inference/learning step seeks to discover underlying expression programs and tissue groups in the data. To accomplish this, we use Markov Chain Monte Carlo (MCMC) sampling [30], an approximate inference method, to estimate the model posterior probability distribution. Each posterior distribution sample describes a configuration of expression programs and tissue groups for the entire dataset; more probable configurations tend to occur in more samples. The final step of the algorithm is model summarization, which produces consensus descriptions of expression programs and tissue groups from the posterior distribution samples. See the Methods section for details.

#### Probability model overview.

**Figure 2:**
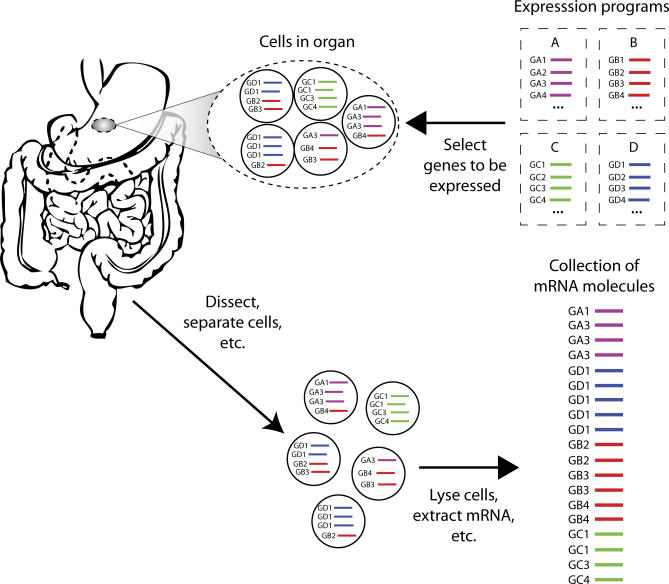
Conceptual Overview of the Data Generation Process for Gene Expression in Human Tissues The GeneProgram probability model can be thought of as a series of “recipes” for constructing the gene expression of tissues, as depicted in this schematic example for a digestive tract. In the upper right, four expression programs (labeled A–D) are shown, consisting of sets of genes (e.g., GA1 represents gene 1 in program A). Cells (circles) throughout the digestive tract choose genes to be expressed probabilistically from the programs. The biological experimenter then collects mRNA by dissecting out the appropriate tissue sample, homogenizing it, lysing cells, and extracting nucleic acids.

The GeneProgram probability model can be understood intuitively as a series of “recipes” for constructing the gene expression of human tissues. Figure 2 presents a schematic diagram of this process, in which we imagine that we are generating the expression data for the digestive tract of a person. The digestive tract is composed of a variety of cell types, with cells of a given type living in different microenvironments, and thus expressing somewhat different sets of genes. We can envision each cell in an organ choosing to express a subset of genes from relevant expression programs; some programs will be shared among many cell types and others will be more specific. As we move along the digestive tract, the cell types present will change, and different expression programs will become active. However, based on the similar physiological functions of the tissues of the digestive tract, we expect more extensive sharing of expression programs than we would between dissimilar organs such as the brain and kidneys. As can be seen in Figure 2, the final steps of our imaginary data generation experiment involve organ dissection, homogenization, cell lysis, and nucleic acid extraction, to yield the total mRNA expressed in the tissue, which is then measured on a DNA microarray.

The conceptual experiment described above for “constructing” collections of mRNA molecules from tissues is analogous to the *topic model,* a probabilistic method developed for information retrieval applications [12,31] and also applied to other domains, such as computer vision [32] and haploinsufficiency profiling [11]. In topic models for information retrieval applications, documents are represented as unordered collections of words, and documents are decomposed into sets of related words called topics that may be shared across documents. In hierarchical versions of such models, documents are further organized into categories, and topics are preferentially shared within the same category. In the GeneProgram model, a unit of mRNA detectable on a microarray is analogous to an individual word in the topic model. Related tissue populations (tissue groups) are analogous to document categories, tissues are analogous to documents, and topics are analogous to expression programs.

GeneProgram extends the hierarchical topic model to capture general patterns of expression changes, such as temporal dynamics, through the novel concept of program *usage modifiers,* which are variables that alter the manner in which each tissue uses an expression program. For instance, for time-series data, usage modifiers would take on values of particular temporal patterns, such as early, middle, or late induction or repression of expression. A tissue then probabilistically chooses a set of genes from a program, and also a value for its usage modifier (e.g., the particular temporal pattern with which to express genes from the program). Because usage modifiers are modeled hierarchically, their values are influenced by all tissues in the data, and especially those in the same group.

GeneProgram handles uncertainty in the numbers of expression programs and tissue groups by using Dirichlet Processes, a non-parametric Bayesian statistical method that provides a prior distribution over the numbers of programs and tissues groups while penalizing model complexity. More specifically, GeneProgram is based on Hierarchical Dirichlet Process mixture models [13], which allow data items to be assigned to groups. Items in the same group preferentially share mixture components, although mixture components may be used across the entire model. We note that in the original Hierarchical Dirichlet Processes formulation [13], items were required to be manually assigned to groups. The GeneProgram model extends this work, automatically determining the number of groups and tissue memberships in the groups.

**Figure 3:**
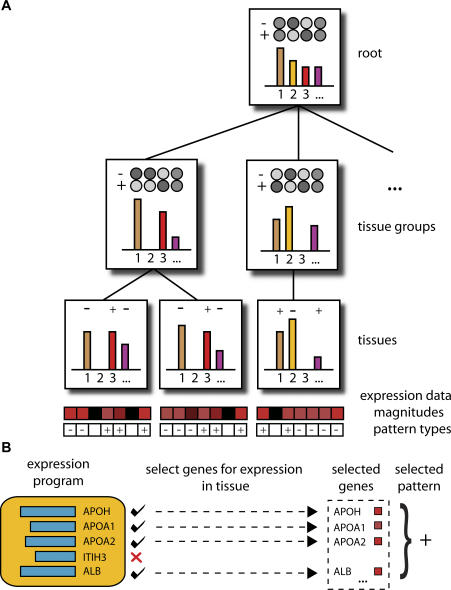
Overview of the GeneProgram Probability Model, Which Is Based on Hierarchical Dirichlet Process Mixture Models (A) The model consists of a three-level hierarchy of Dirichlet Processes. Each node describes a weighted mixture of expression programs (each colored bar represents a different program; heights of bars = mixture weights). The distributions at each level are constructed on the basis of the parent mixtures above. Tissue group and root level nodes maintain distributions over usage modifiers (shaded circles above bars, darker circles = more probable), which are variables that alter the manner in which each tissue uses an expression program. In this example, there are two possible values for usage modifier variables (+ or −), corresponding to gene induction or repression; more complex patterns such as temporal dynamics can be captured by using more values. Tissues are at the leaves of the hierarchy, and choose particular values for usage modifier variables. The observed gene expression in each tissue is characterized by a vector of discretized expression magnitudes (first row of small shaded squares below each tissue) and pattern types (second row of squares with +/− designations below each tissue). (B) Example of a gene expression program, which represents a set of genes that are likely to behave coordinately in particular tissues that use the program. On the left is a simple program containing five genes (colored bars = expression frequencies). A tissue probabilistically chooses a set of genes from a program, and also a setting for its usage modifier variable. Note that usage is consistent across a program for a particular tissue, which facilitates biological interpretation.

The GeneProgram probability model consists of a three-level hierarchy of Dirichlet Processes, as depicted in Figure 3A. Tissue samples are at the lowest level in the hierarchy. Each tissue sample is characterized by a mixture (weighted combination) of expression programs and a set of usage modifiers, which together describe the observed expression magnitudes and patterns of change in the tissue sample. An expression program represents a set of genes that are likely to behave coordinately in particular tissues that use the program, as depicted in Figure 3B. Tissue samples differ in terms of which expression programs they employ, how the programs are weighted, and how the programs are used. The middle level of the hierarchy consists of tissue groups, in which each group represents tissue samples that are similar in their use of expression programs (in terms of both weightings and usage modifiers). The highest and root level in the hierarchy describes a base level mixture of expression programs that is not tissue sample or group specific.

Each node in our hierarchical model maintains a mixture of gene expression programs, and the mixtures at the level below are constructed on the basis of those above. Thus, a tissue sample is decomposed into a collection of gene expression programs, which are potentially shared across the entire model, but are more likely to be shared by related tissues (those in the same tissue group). Further, the usage of each program may differ between tissues, but is more likely to be the same in related tissues. Because our model uses Dirichlet Processes, the numbers of both expression programs and tissue groups are not fixed and may vary with each sample from the model posterior distribution. See Protocol S1 for complete details.

### GeneProgram Accurately Recovered Coherent Gene Sets in Noisy Synthetic Data That Other Algorithms Could Not

**Figure 4:**
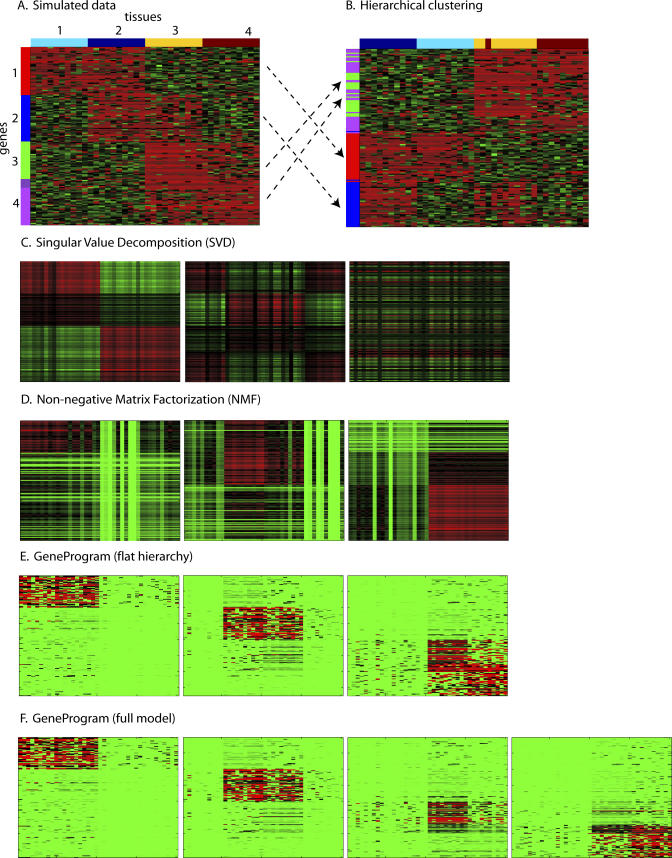
Synthetic Data Experiments Demonstrated GeneProgram's and Other Algorithms' Abilities to Recover Gene Sets from Noisy Data (A) Noisy synthetic data, containing 150 genes (rows) and 40 tissues (columns), with four gene sets and four tissue sample populations. Vertical numbers and colored bars designate gene sets; corresponding horizontal elements designate tissue sample populations. Each gene set contained 40 genes with varying mRNA levels; programs 3 and 4 overlapped in ten genes. See the text for complete details. (B) Hierarchical clustering was applied to sorting both rows (genes) and columns (tissues) of the data. Arrows from (A) indicate resorting of gene sets. Note that hierarchical clustering did not separate gene sets 3 and 4 correctly, and broke up gene set 2 horizontally. (C) SVD factors did not clearly correspond to the distinct gene sets or tissue populations in the synthetic data; the first three factors are shown. (D) An NMF implementation, which searches for the optimal number of factors, was applied to the data. In this case, three factors were optimal. The method performed fairly well in recovering genes sets 1 and 2, although there was some overlap between the sets. However, gene sets 3 and 4 were not recovered as separate sets. (E) A simplified version of GeneProgram using a flat hierarchy (automatic tissue grouping disabled) accurately recovered gene sets 1 and 2, but failed to separate sets 3 and 4. (F) The full GeneProgram implementation using automatic tissue grouping correctly recovered all four gene sets.

We used a simple synthetic data example to explore the kinds of structures GeneProgram and several other well-known unsupervised learning algorithms could recover from noisy data. Our objective with these experiments was to use simulated data to illustrate the capabilities of the algorithms; whether or not particular structures are present in real data can only be answered empirically, and is addressed in the next subsections. In creating synthetic data, we sought to simulate important features of real microarray data profiling human tissues. Thus, we assumed noisy data in which there were several distinct populations of related tissues using different sets of co-expressed genes. In particular, the simulated data contained four sets of co-expressed genes used by 40 tissues divided equally among four tissue populations (see Figure 4A). Each gene set contained 40 genes with varying mRNA levels; gene sets three and four overlapped in ten genes. See the Methods section for details. We note that this scheme for simulating data does not simply recapitulate the assumptions present in the GeneProgram model, because it does not assume discrete and independent “units” of expression signal and it introduces microarray-like noise.

Hierarchical clustering is one of the most frequently used methods for clustering microarray gene expression data. Figure 4B shows the results of hierarchical clustering, using Pearson correlation as a similarity metric and the average linkage method [33], applied to sorting both rows (genes) and columns (tissues) of the synthetic data. As can be seen, hierarchical clustering did reasonably well at sorting tissues and genes independently, although it did not separate gene sets three and four correctly. But, this method's failure to consider genes and tissues simultaneously is known to break up coherent “overhanging” blocks of genes, making interpretation difficult [2,5,8]. This issue was demonstrated in this example by gene sets one and two that “overhang,” thus causing gene set two to be broken up horizontally (blue rows in Figures 4A and 4B).

The inability of hierarchical clustering to handle “overhanging” block structure in data was one of the motivations for the development of biclustering algorithms that take genes and tissues into account simultaneously [2,5,9]. To investigate the behavior of biclustering algorithms, we used Samba, an algorithm that has been shown previously to outperform other biclustering methods [5,34]. Samba produced 23 biclusters from the synthetic data (unpublished data). This method tended to find small subsets of genes co-expressed in some tissues, but did not recover the four gene sets as coherent biclusters. Presumably, this is because Samba does not attempt to incorporate more global constraints on biclusters.

Singular Value Decomposition (SVD) is a matrix factorization method that can be used to approximate a matrix using a smaller number of factors or components. In the context of gene expression data, the method has previously been used to decompose data into “eigengenes” and “eigenexperiments,” linear combinations of genes and experiments, respectively [1,35]. However, it is generally recognized that SVD often produces components that are difficult to interpret [8,36–38]. As can be seen in Figure 4C, components produced by SVD [33] do not clearly correspond to the distinct gene sets or tissue populations in the synthetic data. For instance, the first component is to some extent a composition of gene sets one and three, and subsequent components then add or subtract different combinations of the gene sets.

The development of non-negative matrix factorization (NMF) methods was in part driven by the aforementioned problems with SVD. NMF algorithms decompose a matrix into the product of non-negative matrices [38]. These algorithms generally produce more interpretable factors than does SVD, and have been successfully applied to various problems including gene expression analysis [8,36,37]. Figure 4D shows the application of an NMF-based algorithm to the synthetic data. We used a publicly available implementation [36], which searches for an optimal number of factors using a cophenetic clustering coefficient metric, and in this case we found three factors to be optimal. As can be seen in Figure 4D, NMF did fairly well at recovering gene sets one and two, although there was some overlap between the sets. However, gene sets three and four were indistinguishable.

Figure 4E demonstrates the application of a simplified version of GeneProgram in which tissue groups were not modeled (all tissues were attached to the root of the hierarchy). As can be seen, this version of the algorithm accurately recovered gene sets one and two. However, as with NMF, gene sets three and four completely overlapped.

Figure 4F shows the application of GeneProgram with full automatic learning of tissue groups enabled. As can be seen, the algorithm accurately recovered all four gene sets. By leveraging hierarchical structure in the data, the algorithm had additional information (the pattern of expression program use by related tissues), which presumably allowed it to correctly recover all the synthetic gene sets—something the other methods we tested were not capable of doing.

### GeneProgram Outperformed Biclustering Algorithms in the Discovery of Biologically Relevant Gene Sets in Large Compendia of Mammalian Gene Expression Data

Our objective in this subsection was to apply GeneProgram to large compendia of mammalian gene expression data to compare our method's performance against that of other algorithms. In this regard, we used Novartis Gene Atlas version 2 [20], consisting of genome-wide Affymetrix expression measurements for 79 human and 61 mouse tissues, and a second dataset collected by Shyamsundar et al., consisting of cDNA expression measurements for 115 human tissues [19]. We compared GeneProgram's performance to two biclustering algorithms, Samba [5,34] and a non-negative matrix factorization (NMF) implementation [36]. We chose these two algorithms for comparison because they are popular in the gene expression analysis community, they have previously outperformed other biclustering algorithms, and available implementations are capable of handling large datasets.

Because expression programs characterize both genes and tissues, we used both Gene Ontology (GO) categories [27] and manually derived, broadly physiologically based tissue categories to assess the algorithms' performance. However, GO categories and the manually derived tissue categories represent only limited biological knowledge. So, we were also interested in assessing the consistency of gene sets discovered by each algorithm across the two datasets. Because the two datasets used different microarray platforms and sources for tissues, similarities in discovered gene sets between datasets were likely to be biologically relevant. To analyze gene set consistency for each algorithm, we used the gene sets discovered from one dataset to compute the significance of the overlap with sets produced using the second dataset. We then inverted the analysis and averaged the results to produce correspondence plots. See the Methods section for details.

**Figure 5:**
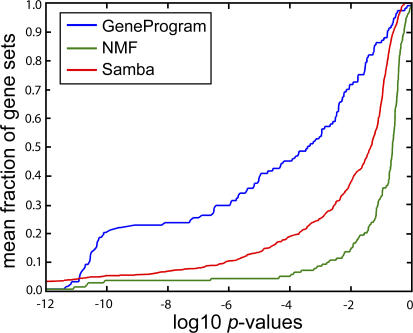
GeneProgram Outperformed Two Popular Biclustering Methods, an NMF Implementation and Samba, in Terms of Gene Set Consistency between Two Large Compendia of Mammalian Tissue Gene Expression Data Because the two data compendia used different microarray platforms and sources for tissues, similarities in discovered gene sets between compendia were likely to be biologically relevant. For each algorithm, we used gene sets discovered from one data compendium to compute the significance of the overlap (*p*-values) with sets produced using the second compendium. We then inverted the analysis and averaged the results to produce the correspondence plots shown. The plots depict log *p*-values on the horizontal axis and the fraction of gene sets with *p*-values below a given value on the vertical axis (see the Methods section for details). The larger fraction of gene sets at most *p*-values suggests that GeneProgram generally produces the most consistent results between the data compendia.

GeneProgram clearly outperformed the other algorithms in both the tissue and gene dimensions on the two datasets considered separately (Table 1), and also in terms of gene set consistency between the datasets (Figure 5). These results suggest several performance trends related to features of the different algorithms. As noted in the section on synthetic data experiments, Samba was successful at finding relatively small sets of genes that are co-expressed in subsets of tissues, but had difficulty uncovering larger structures in data. Presumably, our algorithm's dominance of both Samba and NMF was partly attributable to GeneProgram's hierarchical model. Both Samba and NMF lack such a model, so the assignment of tissues to biclusters was not guided by global relationships among tissues.

**Table 1:**
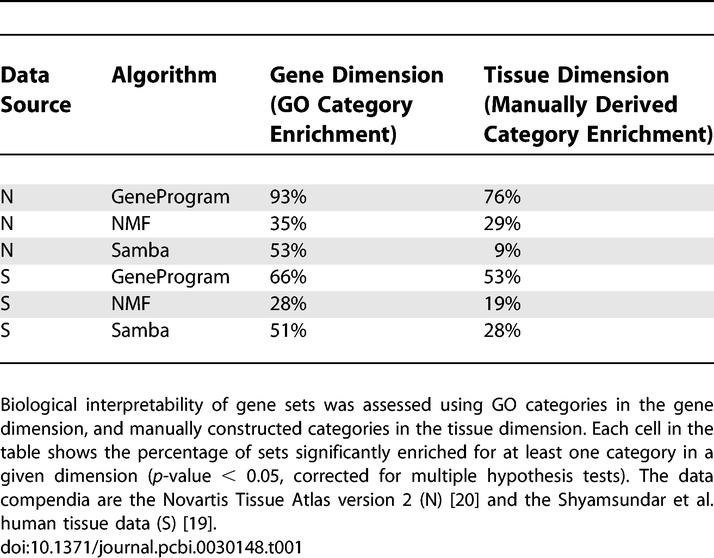
GeneProgram Outperformed Two Popular Biclustering Algorithms, an NMF Implementation and Samba, in Recovering Biologically Interpretable Gene Sets from Two Compendia of Mammalian Gene Expression Experiments

We note also that the algorithms differed substantially in runtimes. For the largest dataset (Novartis Gene Atlas version 2), Samba was fastest (approximately 3 h), GeneProgram the next fastest (approximately 3 d), and NMF the slowest (approximately 6 d), with all software running on a 3.2 GHz Intel Xenon CPU. Although these runtime differences may be attributable in part to implementation details, it is worth noting that GeneProgram, a fully Bayesian model using MCMC sampling for inference, ran faster than the NMF algorithm, which uses a more “traditional” objective maximization algorithm and searches for the appropriate number of biclusters.

### GeneProgram Discovered Five Tissue Groups and 104 Expression Programs in Human Host-Cell Infection Time-Series Data

In the previous two subsections, we used GeneProgram in a manner similar to traditional biclustering algorithms. In this subsection, we take advantage of GeneProgram's ability to find coherent gene sets that may be used with different but consistent temporal dynamics by each tissue sample—a capability not shared with traditional biclustering algorithms.

We applied GeneProgram to a compendium of 62 short time-series gene expression datasets, measuring the responses of human cells to various infectious agents or immune-modulating molecules. See Table S1 for a summary of the data sources used in the compendium and Table S2 for information about each time-series experiment analyzed.

Behavior for each gene over each time-series experiment was mapped to one of six simple temporal patterns. A limited set of possible temporal patterns was intentionally chosen for two reasons. First, in the original studies, a primary feature of interest for all the experiments analyzed was the time of earliest induction or repression of each gene [21,23–25]. Thus, a small set of relevant temporal patterns aids in the biological interpretability of our results. Second, the time-series datasets analyzed had different durations, sampling rates, and numbers of samples. By considering only simple temporal patterns that extract features present in all time-series, we could produce meaningful results spanning all the datasets. The six temporal patterns characterize three temporal phases (early, middle, or late) with two possible directions (induction or repression) for the first significant expression change for each gene in each time-series experiment. See Figure S1 for a summary of the temporal patterns used, and Figure S2 for example genes with expression profiles corresponding to the patterns.

**Figure 6:**
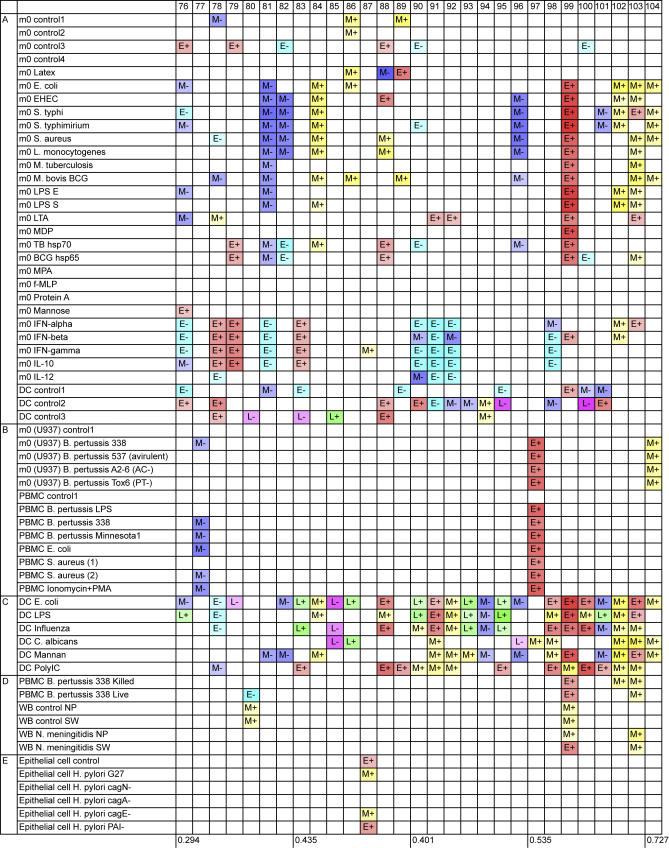
GeneProgram Discovered Five Tissue Groups (A–E) and 104 Expression Programs (Numbered Columns, Only 76–104 Shown Here; 1–75 Shown in Figures S3–S5) in a Compendium of 62 Short Time-Series Gene Expression Datasets Measuring the Responses of Human Cells to Infectious Agents and Immune-Modulating Molecules Each entry in the matrix represents the usage of an expression program (darker shading = higher usage), and matrix entries are colored and labeled with temporal patterns. The patterns are early induction (E+, red), middle induction (M+, yellow), late induction (L+, green), early repression (E−, cyan), middle repression (M−, blue), and late repression (L−, light purple). Generality scores are shown at the bottom of the figure; programs are sorted from lowest to highest scores (left to right).

Figures S3–S5 (programs 1–75) and Figure 6 (programs 76–104) provide graphical summaries and Table S3 provides complete details for the tissue groups and expression programs discovered by GeneProgram from the infection data. The tissue groups essentially corresponded to the different host-cell types used for the infection experiments, although there was some intermingling of the dendritic and peripheral blood mononuclear cell experiments and those using other host-cell types. Expression programs were used by 2–27 experiments (median = 7) and contained 101–410 genes (median = 201). All six temporal patterns were used, although late induction and late repression were used least frequently, likely in part because the corresponding time intervals were the most sparsely sampled in the compendium analyzed. In many cases, usage patterns for a single program were uniformly inductive or repressive, although programs were sometimes used with differently phased patterns by different experiments. However, in other interesting cases, usage patterns were not uniformly inductive or repressive. Specific examples of expression programs with different temporal pattern usage are discussed below.

### Discovered Expression Programs Overlapped Extensively with Key Human Signaling Pathways and Biological Processes

To evaluate the biological relevance of expression programs, we used two external sources of information about gene function: GO biological process categories [27] and Kyoto Encyclopedia of Genes and Genomes (KEGG) pathways [28]. See the Methods section for details.

A large number of expression programs were significantly enriched for GO categories (50%) or KEGG pathways (59%). As expected, many significant GO categories and KEGG pathways were specifically involved with response to infection. Interestingly, a substantial number of significant categories or pathways corresponded to signaling cascades. Further, there were also a number of significantly enriched biological processes or pathways not directly annotated as being infection-related, but that are involved with alterations in cellular physiology consistent with infection. Finally, there were some unexpected significant categories or pathways. See Tables S4 and S5 for details. Specific examples of expression programs enriched for genes involved in key pathways and biological processes are discussed below.

### A Surprising Number of Expression Programs Contained Significant Numbers of Genes Bound by NF-κB Transcription Factor Family Members

We further evaluated the biological relevance of discovered expression programs using genome-wide ChIP–chip data profiling binding of NF-κB family transcription factors in human cell-culture–derived macrophages [29]. NF-κB transcription factors are key controllers of mammalian immune and inflammatory responses [39], so we expected that some genes differentially expressed in the infection time-series compendium would also be bound by NF-κB transcription factors in the ChIP–chip experiments.

Fifteen of the expression programs discovered by GeneProgram were significantly enriched for sets of genes bound by at least one NF-κB family member. This overlap with the binding data was quite large, considering that only 348 genes were bound by NF-κB family members in the ChIP–chip experiments [29]. Overall, the 15 significantly enriched programs tended to be those used by a diverse array of host-cell types exposed to a range of pathogens and their components. This suggests that these expression programs may represent common processes that are strongly induced or repressed during infection via NF-κB family member regulatory activity. Examples of such programs are discussed below.

### The Generality Score Naturally Categorized Programs into a Spectrum of Responses to Infection

We developed a score for assessing the functional generality of expression programs, and demonstrated its utility for automatically characterizing the spectrum of discovered programs—from gene sets involved in response to a specific pathogen in one host-cell type, to those mediating common inflammatory pathways. The *generality score* is the entropy of the normalized distribution of usage of an expression program by all tissues in each tissue group. Because the distribution employed for calculating the score is normalized, tissue groups that only use an expression program a relatively small amount will have little effect on the score. Thus, a high generality score indicates that an expression program is used fairly evenly across many tissue groups; a low score indicates the program is used by tissues from a small number of groups. We note that a score based on expression program usage by individual tissues, rather than by groups, would ignore similarities among tissues and would tend to overestimate the generality of programs if related tissues were present in the dataset. Thus, an informative generality score requires a global organization of tissues into groups, rather than just the local associations of subsets of tissues with individual gene sets provided by biclustering algorithms. Because there is uncertainty in the number of and membership in tissue groups, GeneProgram's Dirichlet Process–based model provides a natural framework for computing the generality score. See Protocol S1 for complete details.

### Programs with Low Generality Scores Were Used by Experiments Spanning a Limited Number of Host-Cell and Infection Types

Experiments involving exposure of gastric epithelial cells to Helicobacter pylori used several low generality expression programs (EPs). This is biologically plausible, because gastric epithelial cells are not involved in principle immune system functions, unlike all other host cells profiled in the dataset [22]. As an example of a program used exclusively by H. pylori–infected epithelial cells, EP 22 (generality = 0.011, five experiments) was enriched for genes involved in regulation of the actin cytoskeleton (KEGG: hsa04810), and was used with a middle induction modifier by all associated experiments. The induction of this pathway is consistent with extensive host-cell shape changes known to occur in H. pylori infection; delayed induction likely reflects the time necessary for bacterial attachment and secretion of proteins that induce host-cell cytoskeletal rearrangements [22].

Peripheral blood mononuclear cell (PBMC) and whole blood experiments also used several low generality programs. This is biologically reasonable, because PBMCs and whole blood represent mixtures of innate and adaptive immunity-mediating cell types, and thus contain cell types not profiled in the other experiments analyzed. Further, the diversity of cell types in PBMC and whole blood cultures allows for critical interactions that are necessary to trigger certain cellular responses [21]. As an example, EP 33 (generality = 0.027, nine experiments) was used by experiments involving PBMCs and whole blood exposed to the Gram-negative bacteria Neisseria meningitides or *Bordetella pertussis,* and was enriched for genes with anti-apoptotic function (GO: 0006916). We hypothesize that this program, which was generally induced in the middle of the time-courses, may involve stabilization of an anti-apoptotic state necessary for maturation and differentiation of peripheral immune cells following infection.

### Programs with Intermediate Generality Scores Were Used by Experiments Involving Host Cells Exposed to a Wider Variety of Agents

Several intermediate generality programs appeared to represent coordinated downregulation of proteolytic and antigen presentation pathways. For example, EP 68 (generality = 0.227, 14 experiments) was used by experiments involving exposure of primary macrophages to a variety of interleukins/interferons, pathogens, or their components. This program was enriched for genes involved in proteasome function (KEGG: hsa03050), and was generally repressed early in the time-series. As another example, EP 77 (generality = 0.298, six experiments) was used by several experiments involving PBMCs or cell-culture–derived macrophages exposed to different bacteria or immune-modulating chemicals. This program was enriched for genes involved in both MHC I and MHC II antigen processing and presentation pathways (KEGG: hsa04612), and was used with a middle repression modifier by all associated experiments. Downregulation of proteasome and antigen presentation pathways subsequent to infection may reflect commitment of phagocytic cells to presentation of antigens from a pathogen that has just been encountered [21].

Several expression programs revealed differences in temporal phasing of the response of host cells exposed to different classes of pathogenic organisms. For example, EP 88 (generality = 0.386, 13 experiments) was enriched for genes involved in ribosomal structure or function (KEGG: hsa03010). Induction of ribosomal genes may be a prelude to production of critical signaling and defensive proteins, such as chemokines and cytokines. EP 88 was induced early in macrophages or dendritic cells exposed to several varieties of Gram-negative bacteria, but induced in the middle of the time-series in host cells exposed to Gram-positive bacteria. As another example, EP 91 (generality = 0.402, 13 experiments), was enriched for genes involved in mRNA splicing (GO: 0000398) and oxidative phosphorylation (KEGG: hsa00190). This program was induced early in time-courses in dendritic cells exposed to bacteria or viruses, but not induced until the middle phase in experiments involving dendritic cells exposed to live fungi or fungal cell components. Interestingly, the program was repressed early in macrophages exposed to various interferons/interleukins. We hypothesize that the phasing differences observed in induction of EPs 88 and 91 may be due to the lesser ability of Gram-positive or fungal organisms to induce critical signaling pathways in innate immune cells. We also note that both programs were significantly enriched for genes bound by NF-κB family members. Interestingly, a number of the NF-κB targets in EP 88 were ribosomal genes, suggesting a direct role for this transcription factor in ribosome activity induction.

Several intermediate generality programs were significantly enriched for surprising signaling pathways or host-cell receptor types. For instance, EP 50 (generality = 0.134, 13 experiments) and EP 84 (generality = 0.331, 12 experiments) were significantly enriched for genes involved in the Wnt signaling pathway (KEGG: hsa04310), including WNT7A and FZD5 in EP 50, and WNT1, WNT5A, and MMP7 in EP 84 (see Figure S6). Wnt signaling pathways have been traditionally implicated in developmental processes [40], and have only recently been shown to be involved in immune system functions [41–43]. For instance, Lobov et al. demonstrated that macrophages can secrete WNT7B, which induces apoptosis in vascular endothelial target cells via the canonical Wnt signaling pathway [42]. Signaling via the WNT5A receptor FZD5 has been implicated in stimulation of pro-inflammatory molecules (e.g., MMP7, TNF-α, IL-12) in macrophages, possibly via both canonical and non-canonical pathways [41,43]. Consistent with these reports of Wnt activity in macrophages, EP 50 was used by macrophages exposed to a variety of bacteria and stimulatory molecules. Interestingly, the program was repressed in macrophages infected with bacteria and induced in cells treated with interleukins or interferons. This difference in program usage may reflect the ability of bacteria to downregulate pro-inflammatory Wnt pathways. In contrast, EP 84, which was mostly used by macrophages and dendritic cells exposed to bacteria or microbial components, was uniformly induced in the middle of the infection time-series. Because the two programs contain different Wnt pathway genes, they may be involved in different inflammatory functions. Further, we note that only some of the Wnt pathway associated genes in EPs 50 and 84 have previously been implicated in macrophage function, making these attractive candidates for future experimental biology work.

EP 55 (generality = 0.161, 12 experiments), which was significantly enriched for genes coding for neurotransmitter or hormonal receptors (KEGG: hsa04080), was another surprising finding. This program was induced in macrophages treated with various interleukins or interferons, and repressed in macrophages exposed to various microbial components. The program contained genes coding for a variety of receptors including those for acetylcholine (CHRM5), cannabinoids (CNR2), dopamine (DRD2), and histamine (HRH1). Although such receptor types are typically found on neurons, they have also been found on macrophages and T cells, and recent studies suggest they may have important pro- and anti-inflammatory properties [44–47]. The different use of this program by macrophages exposed to interleukins/interferons (induction) versus that of those exposed to bacterial components (repression) may reflect bias toward pro- or anti-inflammatory states mediated by different neuroactive receptor signaling pathways.

### Programs with High Generality Scores Were Used by the Full Spectrum of Experiments Involving Exposure of Different Host-Cell Types to a Wide Variety of Agents

Most programs with high generality scores were enriched for genes bound by NF-κB family members and involved in a range of signaling pathways. For instance, EP 99 (generality = 0.585, 27 experiments) appeared to represent a “common infection response” program, which was used by experiments from every tissue group and almost always induced early. This EP contained 203 genes, 15% of which code for cytokines or cytokine receptors, and was also significantly enriched for a number of signaling pathways. Of particular interest, it contained many genes involved in the Toll-like receptor signaling pathway (KEGG: hsa04620, see Figure S7). As expected, many of the genes in the overlap between EP 99 and this pathway code for cytokines, but various genes coding for downstream signaling molecules were also present, including TRAF6, AP-1, NF-κB (p105), and IκBα. Further, EP 99 was significantly enriched for genes bound by NF-κB family members RELB, p65, p50, p52, or c-REL in ChIP–chip experiments. In contrast, EP 102 (generality = 0.634, 15 experiments) was also used by a wide variety of experiments, but was induced in the middle of the time-series of all associated experiments, and was not significantly enriched for binding of any NF-κB family members. This program contained a large number of genes coding for interferons or interferon induced chemokines [18]. Interestingly, many interferon sensitive genes (ISGs) are activated by transcription factors other than NF-κB, and a delay in production of ISGs has previously been noted, presumably due to the time needed to establish critical autocrine and paracrine signaling loops [18].

### Conclusions

We presented a new computational methodology, GeneProgram, specifically designed for analyzing large compendia of human expression data. We demonstrated that GeneProgram produces superior results when compared with traditional biclustering algorithms on two types of tasks. First, we used synthetic data experiments to show that GeneProgram was able to correctly recover gene sets that other popular analysis methods could not. Second, we analyzed two large compendia of mammalian gene expression data from representative normal tissue samples, and demonstrated that GeneProgram outperformed other biclustering methods in the discovery of biologically relevant gene sets.

In our final application, we took advantage of GeneProgram's ability to find coherent gene sets used with different temporal dynamics by each tissue sample—a capability traditional biclustering algorithms do not have. We applied GeneProgram to a compendium of short time-series gene expression datasets exploring the responses of human host cells to infectious agents and immune-modulating molecules. Using this dataset, GeneProgram discovered five tissue groups and 104 expression programs, a substantial number of which were significantly enriched for genes involved in key signaling pathways and/or bound by NF-κB transcription factor family members in ChIP–chip experiments. We introduced an expression program generality score that exploits the tissue groupings automatically learned by GeneProgram, and showed that this score characterizes the functional spectrum of discovered expression programs—from gene sets involved in response to specific pathogens in one host cell type, to those mediating common inflammatory pathways.

GeneProgram automatically discovered many expression programs involved in key pathways related to the response to infection and uncovered temporal phasing differences in program use by some experiments. For instance, programs enriched for genes involved in ribosomal function or energy production were induced earlier in host cells exposed to Gram-negative organisms than in those exposed to Gram-positive organisms or fungi. Some of the discovered programs overlapped with previously described gene sets derived from the same expression data, such as EP 99 and the “common host response” genes discussed by Jenner and Young [18]. However, previous meta-analyses of the data compendium relied on extensive prior biological knowledge and manual inspection of data [18]. In contrast, our method was automatic, discovering expression programs, associating them with consistent temporal patterns, and finding significantly overlapping biological pathways and NF-κB binding from ChIP–chip data.

Some of the gene sets discovered by GeneProgram implicate surprising signaling pathways or host-cell receptor types in the response to infection. In particular, EPs 50 and 84 were significantly enriched for genes involved in the Wnt signaling pathway, and EP 55 was significantly enriched for genes coding for neurotransmitter or hormonal receptors. Wnt signaling pathways [41–43] and neurotransmitter receptors [44–47] have only recently been implicated in the response to infection. To our knowledge, our work is the first to uncover the activity of these pathways in the datasets analyzed, and to characterize the different temporal behaviors of these pathways in response to a variety of infectious agents and immunomodulatory molecules. We believe that the genes in the above-mentioned expression programs constitute particularly attractive candidates for further biological characterization.

GeneProgram encodes assumptions that differ from some previous methods for analyzing expression data and so merit further discussion. First, we model expression data in a semi-quantitative fashion, assuming that discrete levels of mRNA correspond to biologically interpretable expression differences. We believe this is appropriate because popular array technologies can only reliably measure semi-quantitative, relative changes in expression; many relevant consequences of gene expression are threshold phenomena [48–50]; and it is difficult to assign a clear biological interpretation to a full spectrum of continuous expression levels. Second, GeneProgram assumes that discrete “units” of mRNA are independently allocated to expression programs, which captures the phenomena that mRNA transcribed from the same gene may participate in different biological processes throughout a cell or tissue. Independence of mRNA units is an unrealistic assumption, but this approximation, which is important for efficient inference, has worked well in practice for many other applications of topic models [11,12,31]. Finally, GeneProgram handles temporal data by collapsing time-series into predefined, discrete patterns. Overall, we believe that this is a very useful approach for finding interpretable gene expression programs, particularly when analyzing short time-series experiments, in which there are a limited number of clearly meaningful temporal patterns. Further, this method allows us to extract features present in a compendium of time-series, even when the series have different durations, sampling rates, and numbers of samples. In the case of the infection time-series data analyzed in this work, we defined relevant temporal patterns manually, based on prior biological knowledge [21,23–25]. However, temporal patterns could be derived in more automated ways, such as through pre-processing steps that apply time-series clustering algorithms [15,51] to individual series in the data compendium.

Our method produced a large map of human infection–response expression programs with several distinguishing features. First, by simultaneously using information across a variety of host-cell types exposed to a wide range of pathogens and immunomodulatory molecules, GeneProgram was able to dissect the data finely, automatically splitting genes differentially expressed in response to infection among both general and specific programs. Second, because our model explicitly operates on probabilistically ranked gene sets throughout the entire inference process, rather than finding individual differentially expressed genes, our results are more robust to noise. Third, the fact that expression programs provide probabilistically ranked sets of genes also provides a logical means for prioritizing directed biological experiments. Fourth, because our model is fully Bayesian, providing a global penalty for model complexity including for the number of tissue groups and expression programs, the generated map represents a mathematically principled compression of gene expression information throughout the experiments. Finally, although such a large, comprehensive map is inherently complicated, we believe that GeneProgram's automatic grouping of tissues and the associated expression program generality score aid greatly in its interpretation.

GeneProgam is a general method for analyzing large expression data compendium, and we believe that the maps of expression programs discovered by our algorithm will be particularly useful for guiding future biological experiments. Highly specific expression programs can provide candidate genes for diagnostic markers or drug targets that exhibit minimal unintended “cross-talk.” General expression programs may be useful for identifying genes important in regulating and maintaining general biological responses, which may go awry in disease states such as inflammation or malignancy. Additionally, our framework is flexible, and could accommodate other genome-wide sources of biological data in future work, such as DNA-protein binding or DNA sequence motif information. GeneProgram's ability to discover tissue groups and coherent expression programs de novo using a principled probabilistic method, as well as its use of data in a semi-quantitative manner, makes it especially valuable for novel “meta-analysis” applications involving large datasets of unknown complexity in which direct fully quantitative comparisons are difficult.

## Methods

### Datasets and pre-processing.

For our benchmark datasets, we used two data sources. The data from Shyamsundar et al. consisted of 115 human tissue samples obtained from surgeries or autopsies, with expression measured on custom cDNA microarrays [19]. The reference channel on the microarrays consisted of mRNA pooled from 11 established human cell lines. We considered a gene expressed if its ratio was greater than 2.0. The Novartis Gene Atlas version 2 consisted of 79 human and 61 mouse tissue samples, with expression measured on Affymetrix microarrays [20]. We retained pairs of related genes from mouse and human samples using Homologene (build 47) mappings [52]. Arrays were pre-processed using the GC content-adjusted robust multi-array algorithm (GC-RMA) [53]. To correct for probe specific intensity differences, the intensity of each probe was normalized by dividing by its geometric mean in the 31 matched tissues. For genes represented by more than one probe, we used the maximum of the normalized intensities. A gene was considered expressed if its normalized level was greater than 2.0. For both the Novartis Gene Atlas version 2 and Shyamsundar et al. data, we mapped genes to common identifiers using the IDConverter software [54], and retained only the 7,404 genes present in both datasets.

For the infectious disease time-series analysis, we used expression data from six microarray studies [21–26] that had previously been combined and standardized by Jenner and Young [18]. The data used consisted of 347 microarray experiments measuring expression of 5,042 genes in 62 short time-series (see Tables S1 and S2 for summaries of experiments). Behavior for each gene over each time-series experiment was mapped to one of six simple temporal patterns (see Figure S1). Time-points for all experiments were divided into three general phases: early (less than 2 h), middle (greater than 2 h and not more than 12 h), and late (greater than 12 h). For each gene in each time-series experiment, the gene was assigned to the pattern corresponding to the earliest phase in which the gene's expression value exceeded a 2-fold increase (decrease) in at least one experiment in the respective time interval. Further, to be assigned to a pattern, a gene's expression across the time-series was required to be consistent in direction (either a 2-fold increase or decrease in expression but not both). If a gene's expression profile did not meet all these criteria, it was not assigned to any pattern. Seventy-four genes were not assigned to any pattern in any time-series and were thus excluded from further analysis. The absolute expression value for a gene's earliest induction (repression) in each time-series was then used to represent the magnitude of differential expression.

Expression data magnitudes were discretized into three levels using a mutual information-based greedy agglomerative merging algorithm, as described in Hartemink et al. [55]. In brief, continuous expression levels were first uniformly discretized into a large number of levels. The algorithm then repeatedly found the best two adjacent levels to merge by minimizing the reduction in the pairwise mutual information between all expression vectors. The appropriate number of levels to stop at was determined by choosing the inflection point on the curve obtained by plotting the score against the number of levels. We obtained three levels for all the datasets. To analyze the sensitivity of our results to the number of discretization levels, we created correspondence plots for the largest dataset (the Novartis Gene Atlas version 2) using two, three, and four discretization levels (see Figure S8). As can be seen, at all the discretization levels examined, GeneProgram outperformed the other biclustering methods, and fluctuations across results at different numbers of discretization levels were considerably smaller than differences between GeneProgram and the other algorithms.

To validate expression programs discovered in the infectious disease data, we used genome-wide ChIP–chip data profiling binding of five transcription factors in the NF-κB family in untreated or lipopolysaccharide (LPS) stimulated cell-culture–derived human macrophages [29]. The experimenters obtained data before and after 1 h of LPS stimulation, and binding was measured using microarrays with probes covering proximal promoters of 9,492 genes. We used the same criteria for determining binding events as in the original study (a *p*-value threshold of 0.002).

### Probability model.

The GeneProgram model is an extension of the Hierarchical Dirichlet Process mixture model as described in Teh et al. [13]. In Hierarchical Dirichlet Processes, dependencies are specified among a set of Dirichlet Processes by arranging them in a tree structure. At each level in the tree, the base distribution for the Dirichlet Process is a sample from the parent Dirichlet Process above. In GeneProgram, each expression program corresponds to a mixture component in the Hierarchical Dirichlet Process model. Because the model is hierarchical, expression programs are shared by all Dirichlet Processes in the model. An expression program specifies a multinomial distribution over genes, and a set of usage modifier variables (one for each tissue). Discrete expression levels are treated analogously to word occurrences in document-topic models. Thus, a tissue's vector of gene expression levels is converted into a collection of expression events, in which the number of events for a given gene equals the discrete expression level of that gene in the tissue. A pattern type (e.g., early induction) is associated with each expression event. The model assumes that each gene expression event in a tissue is independently generated by an expression program. One usage modifier variable is associated with each tissue for each program, and thus specifies the possible pattern type for genes from the program used by the tissue. Usage variables are sampled hierarchically, and thus influenced by other tissues. In the original Hierarchical Dirichlet Process formulation [13], the entire tree structure was assumed to be pre-specified. We extend this work, by allowing the model to learn the number of groups and the memberships of tissues in these groups. Thus, groups themselves are generated by a Dirichlet Process, which uses samples from the root of the Hierarchical Dirichlet Process as the base distribution. See Protocol S1 for complete details.

### Model inference and summarization.

The posterior distribution for the model is approximated via MCMC sampling [30]. Our inference method is based on the technique described for efficient MCMC sampling in Hierarchical Dirichlet Processes [13], with additional steps added for sampling the tissue group assignments and usage variables. See Protocol S1 for complete details.

The final step of the GeneProgram algorithm summarizes the approximated model posterior probability distribution with *consensus tissue groups* and *recurrent expression programs*. The posterior distributions of Dirichlet Process mixture models are particularly challenging to summarize because the number of mixture components may differ for each sample. Previous approaches for summarizing Dirichlet Process mixture model components have used pairwise co-clustering probabilities as a similarity measure for input into an agglomerative clustering algorithm [14]. This method is feasible if there are a relatively small number of items to be clustered, and we employ it for producing consensus tissue groups. Consensus tissue groups are constructed by first computing the empirical probability that a pair of tissues will be assigned to the same tissue group. The empirical co-grouping probabilities are then used as pairwise similarity measures in a standard bottom-up agglomerative hierarchical clustering algorithm using complete linkage [56]. See Protocol S1 for complete details. However, this method is not feasible for summarizing expression programs in large datasets because of the number of pairwise probabilities that would need to be calculated for each sample.

We developed a novel method for summarization of the model posterior distribution, which discovers recurrent expression programs by combining information from similar expression programs that reoccur across multiple MCMC model posterior samples. Each expression program in each sample is compared against recurrent programs derived from previous samples. If the Hellinger distance between a program's distribution of gene occurrences and that of a recurrent program is sufficiently small, the programs are merged; otherwise, a new recurrent program is instantiated. Statistics are tracked for each recurrent expression program, including the number of posterior samples it occurs in, its average usage by tissues, and average expression levels of genes in the program. After all recurrent expression programs have been collected, infrequently occurring programs are filtered out. See Protocol S1 for complete details.

### Synthetic data.

We generated four synthetic gene sets used by 40 tissue samples divided equally among four tissue populations. Each gene set contained 40 genes with varying mRNA levels; gene sets three and four overlapped in ten genes. Each tissue population specifies the mean number of genes from each gene set used by tissue samples in the population. For each tissue sample, the number of genes used from each gene set was sampled from the population mean, and then genes were picked from each gene set with a probability weighted by the gene's underlying mRNA level. Finally, the mRNA level for each gene was perturbed by additive and multiplicative noise to simulate microarray noise. See Protocol S1 for complete details.

### Tissue and gene dimension category enrichment analysis and correspondence plots.

We manually classified tissues from Novartis Gene Atlas version 2 and Shyamsundar et al. datasets into ten and eight high-level, physiologically based categories respectively (see Tables S6 and S7).

We mapped genes to GO biological process categories [27] and KEGG pathways [28] using the IDConverter software [54]. For calculating enrichment scores, we considered human GO categories or KEGG pathways containing between five and 200 genes.

For determining enrichment significance of GO categories, KEGG pathways, or NF-κB family transcription factor binding, we used the hypergeometric distribution to compute *p*-values. We corrected for multiple hypothesis tests using the procedure of Benjamini and Hochberg [57]. We used a false-discovery rate cutoff of 0.05.

Correspondence plots have previously been used for sensitive, graphical comparisons of biclustering algorithm performance [5]. These plots depict log *p*-values on the horizontal axis and the fraction of biclusters with *p*-values below a given value on the vertical axis. Depicted *p*-values are from the most abundant class for each bicluster (i.e., that with the largest number of genes or tissue in the overlap) and calculated using the hypergeometric distribution. Note that biclusters with large *p*-values are not significantly enriched for any class, and may represent noise.

### Software availability.

Complete Java source code is available upon request.

## Supporting Information

Figure S1 Schematic of Six Pre-Defined Temporal Patterns Used in Analyzing the Infection Time-Series Compendium (133 KB JPG)

Figure S2 Examples of Genes from the Infection Time-Series Compendium That Match the Six Pre-Defined Temporal Patterns (312 KB JPG)

Figure S3 Expression Programs 1–25 Discovered in the Infection Time-Series Compendium See Figure 6 for figure legend.
(1.0 MB JPG)

Figure S4 Expression Programs 26–50 Discovered in the Infection Time-Series Compendium See Figure 6 for figure legend.
(1.1 MB JPG)

Figure S5 Expression Programs 51–75 Discovered in the Infection Time-Series Compendium See Figure 6 for figure legend.
(1.2 MB JPG)

Figure S6 Overview of the Wnt Signaling Pathways and Relevant Genes in EP 50 (Pink Shading) and EP 84 (Green Shading); Adapted from the KEGG Pathway Graphic (564 KB JPG)

Figure S7 Overview of the Toll-Like Receptor Signaling Pathways and Relevant Genes in EP 99 (Pink Shading); Adapted from the KEGG Pathway Graphic (481 KB JPG)

Figure S8 Correspondence Plots for Gene and Tissue Dimensions from Analysis of Novartis Gene Atlas Version 2 Using Samba, NMF, and Two, Three, and Four Discretization Levels in GeneProgram (277 KB JPG)

Protocol S1 Detailed Descriptions of GeneProgram Probability Model, Inference Methods, and an Overview of Dirichlet Processes (607 KB PDF)

Table S1 Summary of Data Sources Used in the Infection Time-Series Compendium (73 KB PDF)

Table S2 Summary of Individual Experiments in the Infection Time-Series Compendium (26 KB XLS)

Table S3 Details for All 104 Expression Programs Discovered in the Infection Time-Series Compendium (1.8 MB XLS)

Table S4 Summary of Selected GO Biological Process Categories and KEGG Pathways for Which Expression Programs Discovered in the Infection Time-Series Compendium Were Significantly Enriched (65 KB PDF)

Table S5 Complete List of GO Biological Process Categories and KEGG Pathways for Which Expression Programs Discovered in the Infection Time-Series Compendium Were Significantly Enriched (57 KB XLS)

Table S6 Manual Classification of Novartis Gene Atlas Version 2 [20] Tissue Samples into Ten High-Level, Physiologically Based Categories (12 KB PDF)

Table S7 Manual Classification of Tissue Samples from the Shyamsundar et al. [19] Data Compendium into Eight High-Level, Physiologically Based Categories (12 KB PDF)

## Abbreviations

ChIP: chromatin immunoprecipitation
EP: expression program
GO: gene ontology
KEGG: Kyoto Encyclopedia of Genes and Genomes
MCMC: Markov chain Monte Carlo
NMF: non-negative matrix factorization
PBMC: peripheral blood mononuclear cell
SVD: singular value decomposition
