# Supplementary material for: Automated Discovery of Functional Generality of Human Gene Expression Programs
Source: PLoS Comput Biol. 2007 Aug 10;3(8):e148. doi: 10.1371/journal.pcbi.0030148 (PMC1941755; doi:10.1371/journal.pcbi.0030148)
Supplement: Supplementary file 1 [file pcbi.0030148.sd001.pdf]

CHAPTER 1

GeneProgram Protocol S1

1.1 The GeneProgram probability model

1.1.1 Overview

The GeneProgram probability model is an extension of the Hierarchical Dirichlet Process mixture model as described in Teh *et al.* [21]. See the appendix of this document for further details on Dirichlet Processes. Briefly, a Dirichlet Process (DP) is characterized by two parameters, a base probability distribution and a concentration or scaling parameter. It has been shown that with probability one, each random sample from a DP is a discrete infinite mixture over samples from the base distribution [20]. The number of "concentrated" mixture components (i.e., those with large weights) is controlled by the scaling or concentration parameter. A mixture model over data is constructed by using each component of the sample from the DP to parameterize the likelihood for generating the data. For instance, an infinite Gaussian mixture model is constructed by using a base distribution that is a prior over mean and variance. In Hierarchical Dirichlet Processes (HDPs), dependencies are specified among a set of DPs by arranging them in a tree structure. At each level in the tree, the base distribution for the DP is a sample from the parent DP above.

The GeneProgram model consists of three levels of DPs. Starting from the leaves these are: tissues, tissue groups, and the root. Each expression program corresponds to a mixture component of the HDP. Because the model is hierarchical, the expression programs are shared by all DPs in the model. An expression program specifies a multinomial distribution over genes, and a set of usage modifier variables (one for each tissue). Discrete expression levels are treated analogously to word occurrences in document-topic models. Thus, a tissue's vector of gene expression levels is converted into a collection of expression events, in which the number of events for a given gene equals the discrete expression level of that gene in the tissue. A pattern type

(e.g., induction or repression) is associated with each expression event. The model assumes that each gene expression event in a tissue is independently generated by an expression program. One usage modifier variable is associated with each tissue for each program, and thus specifies the pattern type for genes from the program used by the tissue. Usage modifiers are modeled hierarchically, and so their values are influenced by all tissues in the data, and especially those in the same group.

In the original HDP formulation [21], the entire tree structure was assumed to be pre-specified. We extend this work, by allowing the model to learn the number of groups and the memberships of tissues in these groups. Thus, groups themselves are generated by a DP, which uses samples from the root process DP as its base distribution.

1.1.2 Formal model description

Figure 1-1 depicts the model using graphical model notation with plates and Table 1.1 summarizes the random variables in the model.

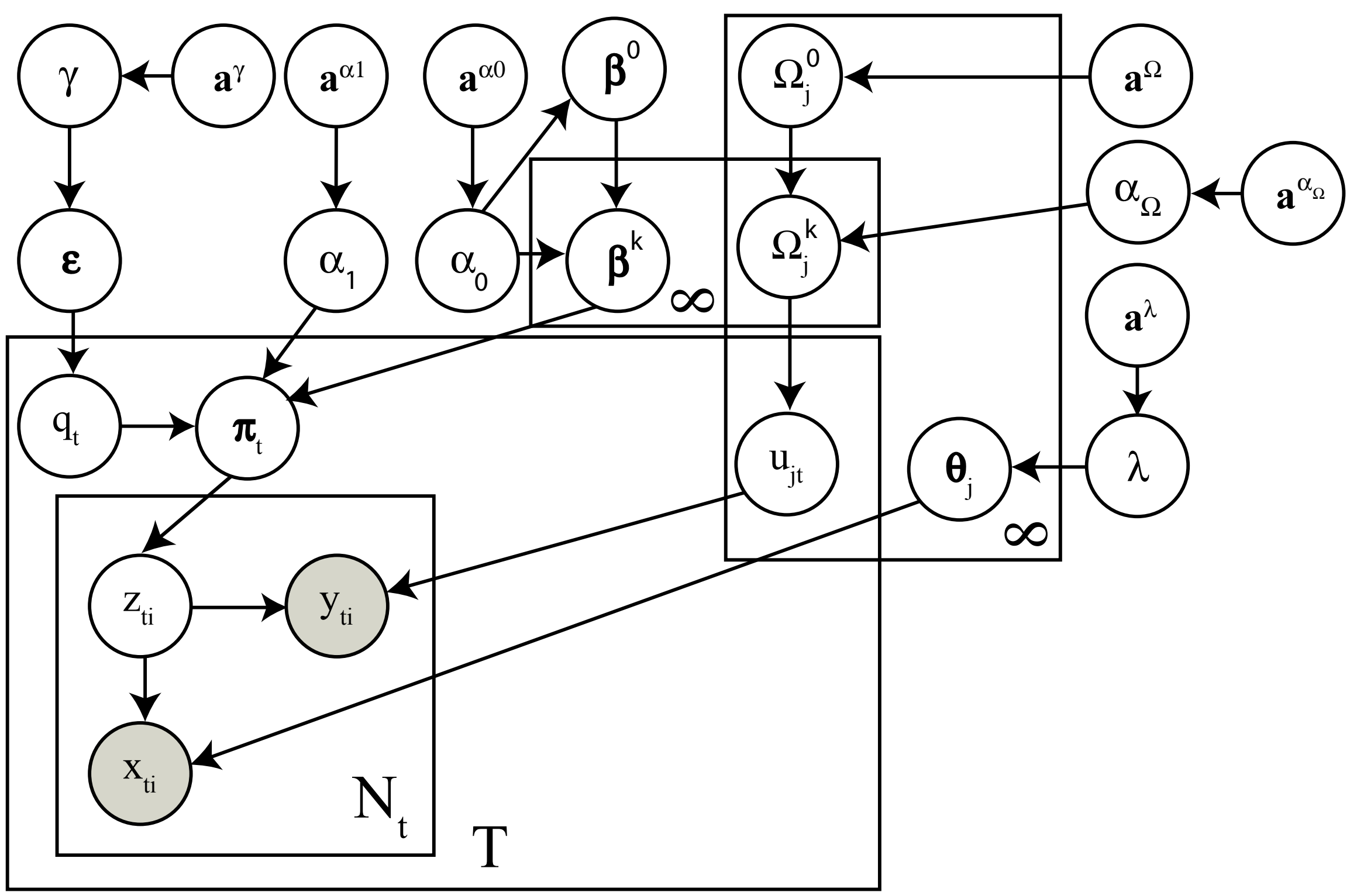

Figure 1-1: The GeneProgram model is depicted using graphical model notation with plates. Circles represent variables, and arrows denote dependencies among variables. Vectors are depicted with bold type, and observed variables are shown inside shaded circles. Rectangles represent plates, or repeated sub-structures in the model. See the text and Table 1.1 for details.

Var.	Dim.	Description	Cond. distribution or prior
x_{ti}	1	Expression event i in tissue t; corresponds directly to observed data.	Multinomial, given the assignment to expression program j.
y_{ti}	1	Pattern type for expression event i in tissue t.	Multinomial, given the assignment to expression program j and selection of the program usage by the tissue.
z_{ti}	1	Assignment variable of an expression event to an expression program, i.e., $\mathsf{z}_{ti} = j$ indicates that expression event i in tissue t is assigned to expression program j.	Generated from mixing probabilities over expression programs for the tissue, i.e., $\mathrm{p}(\mathsf{z}_{ti} = j \mid \boldsymbol{\pi}_t) = \pi_{tj}$.
$\boldsymbol{\pi}_t$	∞	Mixing probabilities over expression programs for tissue t.	DP, given its parent mixing probabilities and concentration parameters, i.e., $\boldsymbol{\pi}_t \mid \alpha_1, \boldsymbol{\beta}^0 \sim \mathrm{DP}(\alpha_1, \boldsymbol{\beta}^0)$.
$\boldsymbol{\beta}^k$	∞	Mixing probabilities over expression programs at the level of tissue group k; middle level in the DP hierarchy.	DP, given its parent mixing probabilities and concentration parameters, i.e., $\boldsymbol{\beta}^k \mid \alpha_0, \boldsymbol{\beta}^0 \sim \mathrm{DP}(\alpha_0, \boldsymbol{\beta}^0)$.
$\boldsymbol{\beta}^0$	∞	Root level mixing probabilities in the DP heterarchy.	DP, generated from the stick-breaking distribution given its concentration parameter, i.e., $\boldsymbol{\beta}^0 \mid \alpha_0 \sim \mathrm{Stick}(\alpha_0)$.
$\boldsymbol{\theta}_j$	M	Parameters for expression, program j, describing a multinomial distribution over genes.	Dirichlet distribution prior (parameterized by λ).
λ	1	Pseudo-count parameter for a symmetric Dirichlet distribution.	Gamma distribution prior with a two-dimensional hyperparameter vector $\boldsymbol{a}^{\lambda}$.
u_{jt}	1	Usage modifier variable for expression program j by tissue t.	Multinomial, given the tissue group k level shared parameters, i.e., $\mathsf{u}_{jt} \mid \boldsymbol{\Omega}_j^k, \mathsf{q}_t = k \sim \mathrm{Multinomial}(\boldsymbol{\Omega}_j^k)$.
$\boldsymbol{\Omega}_j^k$	V	Tissue group k level parameters for usage modifiers of expression program j.	Dirichlet distribution prior, i.e., $\boldsymbol{\Omega}_j^k \sim \mathrm{Dirichlet}(\alpha_\Omega \Omega_{j1}^0, \ldots, \alpha_\Omega \Omega_{jV}^0)$.
$\boldsymbol{\Omega}_j^0$	V	Root level parameters for usage modifiers of expression program j.	Dirichlet distribution prior (parameterized by $\boldsymbol{a}^{\Omega}$).
q_t	1	Assignment variable of tissues to groups, i.e., $\mathsf{q}_t = k$ indicates that tissue t belongs to tissue group k.	Generated from mixing probabilities over tissue groups, i.e, $\mathrm{p}(\mathsf{q}_t = k \mid \boldsymbol{\epsilon}) = \epsilon_k$.
$\boldsymbol{\epsilon}$	∞	Mixing probabilities over the tissue groups.	DP, generated from the stick-breaking prior given its concentration parameter, i.e, $\boldsymbol{\epsilon} \mid \gamma \sim \mathrm{Stick}(\gamma)$.
α_1	1	Concentration parameter for $\boldsymbol{\pi}_t$.	Gamma distribution prior with two-dimensional hyperparameter vector $\boldsymbol{a}^{\alpha_1}$.
α_0	1	Concentration parameter for $\boldsymbol{\beta}^0$ and $\boldsymbol{\beta}^k$.	Gamma distribution prior with two-dimensional hyperparameter vector $\boldsymbol{a}^{\alpha_0}$.
γ	1	Concentration parameter for $\boldsymbol{\epsilon}$.	Gamma distribution prior with two-dimensional hyperparameter vector $\boldsymbol{a}^{\gamma}$.
α_Ω	1	Concentration parameter for $\boldsymbol{\Omega}_j^k$.	Gamma distribution prior with two-dimensional hyperparameter vector $\boldsymbol{a}^{\alpha_\Omega}$.

Table 1.1: Summary of random variables used in the GeneProgram model. The columns are: variable name (vectors are in bold type), dimensions of the variable, description, and the conditional or prior distribution on the variable.

We will begin by describing the model at the level of observed data, and then move up the hierarchy. Assume that there are T tissues and G genes. The expression data associated with each tissue t consists of a G-dimensional vector $\mathbf{e}_t$ of discrete expression levels, i.e., $\mathsf{e}_{tg} \in \{0, 1, \ldots, E\}$ is the expression level of gene g in tissue t, where there are E possible discrete expression levels.

A tissue's vector of gene expression levels is converted into a collection of expression events, in which the number of events for a given gene equals the expression level of that gene in the tissue. This representation of expression levels as an unordered "bag of expression events" is analogous to the representation of words in a document as a "bag of words" in topic models. To be precise, let $\mathbf{x}_t$ denote a set of expression events for tissue t, and define a mapping ω from $\mathbf{x}_t$ to genes, where $\omega(\mathsf{x}_{ti}) = g$ iff $\mathsf{e}_{tg} > 0$. The vector $\mathbf{x}_t$ will have N_t elements, where $N_t = \sum_{g=1}^{G} e_{tg}$, i.e., as many elements as there are discrete expression events in the tissue.

We associate an observed pattern type with each expression event. The pattern type, denoted by y_{ti}, can take one of V values. For instance, if we are modeling induction and repression, $V = 2$ and $\mathsf{y}_{ti} \in \{-1, 1\}$, representing the direction of expression change for the gene.

The model assumes that each gene expression event in a tissue is independently generated by an expression program. The variable z_{ti} assigns gene expression events to programs, i.e., $\mathsf{z}_{ti} = j$ indicates that x_{ti} was generated from the jth expression program. An expression program specifies a multinomial probability distribution over genes. To be precise, let $\boldsymbol{\theta}_j$ represent a parameter vector of size G for expression program j. Then, the probability of generating expression event x_{ti} corresponding to gene g given that it is assigned to expression program j is $\mathrm{p}(\omega(\mathsf{x}_{ti}) = g \mid \mathsf{z}_{ti} = j, \boldsymbol{\theta}_j) = \theta_{jg}$. We use a symmetric Dirichlet prior for $\boldsymbol{\theta}_j$ with parameter λ, and a Gamma prior for λ with hyperparameter vector $\boldsymbol{a}^{\lambda}$.

Program usage modifier variables influence which pattern types are generated for genes in an expression program used by a specific tissue. We denote the usage modifier variable for tissue t using expression program j by u_{jt}, where u_{jt} can take on one of V values. Usage modifier variables influence how pattern types are generated through a compatibility function $\psi(\cdot, \cdot)$, which simply specifies the probability of generating a particular observed pattern type given some usage modifier value, i.e., $\psi(y_{ti}, u_{jt}) = \mathrm{p}(\mathsf{y}_{ti} \mid \mathsf{u}_{jt})$. As an example, if we are modeling induction and repression, we might specify a symmetrical compatibility function that returns a large probability when the usage modifier and pattern type variables take on the same value, and a small probability otherwise, i.e., $\psi(-1, -1) = \psi(1, 1) = 0.99$ and $\psi(-1, 1) = \psi(1, -1) = 0.01$.

Usage modifier variables themselves are generated via multinomial distributions parameterized by expression program level parameters, where we have a separate set of such V-dimensional parameters, $\boldsymbol{\Omega}_j^k$ for each expression program j and tissue group k. For each expression program j, these parameters share a common top-level Dirichlet prior parameterized by $\boldsymbol{\Omega}_j^0$ and α_Ω. That is, $\boldsymbol{\Omega}_j^k \sim \mathrm{Dirichlet}(\alpha_\Omega \Omega_{j1}^0, \ldots, \alpha_\Omega \Omega_{jV}^0)$. We assume that α_Ω has a Gamma prior with hyperparameter vector $\boldsymbol{a}^{\alpha_\Omega}$. Further, we have that $\boldsymbol{\Omega}_j^0 \sim \mathrm{Dirichlet}(a_1^\Omega, \ldots, a_V^\Omega)$, where $\boldsymbol{a}^\Omega$ is a V-dimensional vector of hyperparameters.

The mixing probabilities over expression programs are generated by the DPs in the hierarchy. To be precise, let $\boldsymbol{\pi}_t$ denote the mixing probabilities at the leaf level in the DP hierarchy. That is, $\boldsymbol{\pi}_t$ denotes the mixing probabilities over expression programs for tissue t, i.e., $\mathrm{p}(\mathsf{z}_{ti} = j \mid \boldsymbol{\pi}_t) = \pi_{tj}$. Let $\boldsymbol{\beta}^k$ denote the mixing probabilities at the middle level in the DP hiearchy. That is, $\boldsymbol{\beta}^k$ denotes the mixing probabilities over expression programs at the level of tissue group k. Finally, we let $\boldsymbol{\beta}^0$ denote the root-level mixing probabilities. In the stick-breaking construction for HDP models, it is assumed that root level mixing probabilities are generated by the stick-breaking distribution, i.e., $\boldsymbol{\beta}^0 \mid \alpha_0 \sim \mathrm{Stick}(\alpha_0)$, where $\alpha_0 \sim \mathrm{Gamma}(\boldsymbol{a}^{\alpha_0})$. The hierarchical structure of the model then implies that $\boldsymbol{\beta}^k$ is conditionally distributed as a Dirichlet Process, i.e., $\boldsymbol{\beta}^k \mid \alpha_0, \boldsymbol{\beta}^0 \sim \mathrm{DP}(\alpha_0, \boldsymbol{\beta}^0)$, where we assume that $\boldsymbol{\beta}^k$ also uses concentration parameter α_0.

The tissue level expression program mixing probabilities $\boldsymbol{\pi}_t$ depend on the group that the tissue is assigned to. The variable q_t assigns tissues to groups, i.e., $\mathsf{q}_t = k$ indicates that tissue t belongs to tissue group k and $\mathrm{p}(\mathsf{q}_t = k \mid \boldsymbol{\epsilon}) = \epsilon_k$, where $\boldsymbol{\epsilon}$ represents mixing probabilities over the tissue groups. The mixing probabilities ϵ_k over tissue groups are also modeled using a Dirichlet Process. That is, $\boldsymbol{\epsilon} \mid \gamma \sim \mathrm{Stick}(\gamma)$, where γ is a concentration parameter with $\gamma \sim \mathrm{Gamma}(\boldsymbol{a}^{\gamma})$. Given an assignment of tissue t to group k, the tissue level mixing probabilities over expression programs $\boldsymbol{\pi}_t$ are then generated from the middle level mixing probabilities $\boldsymbol{\beta}^k$. That is, $\boldsymbol{\pi}_t \mid \mathsf{q}_t = k, \alpha_1, \boldsymbol{\beta}^k \sim \mathrm{DP}(\alpha_1, \boldsymbol{\beta}^k)$, where α_1 is a concentration parameter with hyperparameters $\boldsymbol{a}^{\alpha_1}$, i.e., $\alpha_1 \sim \mathrm{Gamma}(\boldsymbol{a}^{\alpha_1})$. This completes our formal description of the GeneProgram probability model.

1.2 Model inference

The posterior distribution for the model is approximated via Markov Chain Monte Carlo (MCMC) sampling using the follow steps:

1. Sample each assignment of an expression event to an expression program, z_{ti}; create new expression programs as necessary.
2. Sample each usage modifier variable u_{jt} for each tissue and expression program.
3. Sample $\boldsymbol{\beta}^0$, $\boldsymbol{\beta}^k$ and auxiliary variables for all tissue groups.
4. Sample tissue group assignments q_t for all tissues; create new tissue groups as necessary.
5. Sample $\boldsymbol{\Omega}_j^k$ and auxiliary variables for each expression program and tissue group.
6. Sample $\boldsymbol{\Omega}_j^0$ and auxiliary variables for each expression program.
7. Sample concentration parameters α_0, α_1 and γ.
8. Sample expression program Dirichlet prior parameter λ.

9. Sample concentration parameter α_Ω.

Note that $\mathsf{x}_{ti} \mid \mathsf{z}_{ti} = j, \boldsymbol{\theta}_j \sim \text{Multinomial}(\boldsymbol{\theta}_j)$, and $\boldsymbol{\theta}_j$ is Dirichlet distributed, allowing us to integrate out $\boldsymbol{\theta}_j$ when computing the posterior for z_{ti}. This means that we do not need to represent $\boldsymbol{\theta}_j$ explicitly during sampling.

Steps 3 and 7 are identical to those described by Teh *et al.* in their auxiliary variable sampling scheme [21] (see Section A.3 for further details). Step 8 uses the auxiliary variable sampling method for resampling the parameter for a symmetric Dirichlet prior, as detailed in [6].

In step 1, we sample z_{ti}, the assignment of expression event i in tissue t to an expression program. For assignment to a non-empty expression program j, the conditional distribution for z_{ti} is given by:

$$\mathrm{p}(\mathsf{z}_{ti} = j \mid \mathbf{z}_{-ti}, \boldsymbol{\beta}, \boldsymbol{\theta}, \boldsymbol{\Omega}, \mathbf{u}, \mathbf{x}, \mathbf{y}, \mathbf{q}_{-t}, \mathsf{q}_t = k) \propto (\alpha_1 \beta_j^k + n_{tj}^{-i}) F(\mathsf{x}_{ti} \mid \theta_j) \psi(y_{ti}, u_{jt}) \mathrm{p}(\mathsf{u}_{jt} \mid \boldsymbol{\Omega}_j^k)$$

Here, $\mathbf{z}_{-ti}$ denotes the assignments of all expression events excluding event i in tissue t, n_{tj}^{-i} denotes the number of events from tissue t assigned to program j excluding event i, $F(\cdot)$ denotes the mixture component likelihood (multinomial), and $\psi(\cdot, \cdot)$ is the compatibility function defined in Section 1.1.2.

For assignment to a new expression program, the conditional distribution for z_{ti} is given by:

$$\mathrm{p}(\mathsf{z}_{ti} \neq \mathsf{z}_{tl} \; \forall \, t, l \neq i \mid \mathbf{z}_{-ti}, \boldsymbol{\beta}, \boldsymbol{\theta}, \boldsymbol{\Omega}, \mathbf{u}, \mathbf{x}, \mathbf{y}, \mathbf{q}_{-t}, \mathsf{q}_t = k) \propto \\ (\alpha_1 \beta_*^0) \int F(\mathsf{x}_{ti} \mid \xi) H(\xi) d\xi \left(\sum_{v=1}^{V} \Omega_{*v} \psi(y_{ti}, v) \right)$$

Here, $H(\cdot)$ is the prior for the mixture component likelihood, β_*^0 represents the mixture weight for the new component and $\boldsymbol{\Omega}_*$ the respective new parameter values. The new weight is sampled as described in Teh *et al.* [21] (see Section A.3). The new $\boldsymbol{\Omega}_*$ is simply sampled from its prior, i.e., $\boldsymbol{\Omega}_* \sim \text{Dirichlet}(\boldsymbol{a}^\Omega)$.

In step 2, The usage modifier variables u_{jt} for each tissue t and expression program j are sampled. The posterior distribution for these variables is given by:

$$\mathrm{p}(\mathsf{u}_{jt} = v \mid \mathbf{z}, \boldsymbol{\Omega}, \mathbf{y}, \mathbf{q}_{-t}, \mathsf{q}_t = k) \propto \Omega_{jv}^k \prod_{i=1}^{N_t} \psi(y_{ti}, v)$$

Here, N_t is the number of expression events in tissue t.

In step 4, we must compute the posteriors for tissue group assignments. This can be written as:

$$\mathrm{p}(\mathsf{q}_t = k \mid \mathbf{z}_t, \mathsf{q}_{-t}, \mathbf{y}, \alpha_0, \gamma, \boldsymbol{\beta}^k, \boldsymbol{\Omega}^0, \boldsymbol{\Omega}^k) \propto \\ \mathrm{p}(\mathsf{q}_t = k \mid \mathsf{q}_{-t}, \gamma) \prod_{i=1}^{N_t} \int \mathrm{p}(\mathsf{z}_{ti} \mid \boldsymbol{\pi}_t) \mathrm{p}(\boldsymbol{\pi}_t \mid \boldsymbol{\beta}^k, \alpha_0) d\boldsymbol{\pi}_t \left(\sum_{j=1}^{J} \sum_{i=1}^{N_t} \sum_{v=1}^{V} \Omega_{jv}^k \psi(y_{ti}, v) \right)$$

Here, $\mathbf{q}_{-t}$ denotes all tissue group assignments excluding tissue t, and J is the number of non-empty expression programs. Note that because the conditional distributions for z_{ti} and $\boldsymbol{\pi}_t$ are conjugate, the integral in the above equation can be computed in closed form. If a tissue is assigned to a new group, we must also sample new parameters, i.e., $\boldsymbol{\Omega}_j^* \sim \text{Dirichlet}(\alpha_\Omega \Omega_{j1}^0, \ldots, \alpha_\Omega \Omega_{jV}^0)$ for $j = 1, ..., J$.

In step 5, we need to sample from the posterior distributions for the tissue group level usage modifier variable priors, $\boldsymbol{\Omega}_j^k$. Because the prior for $\boldsymbol{\Omega}_j^k$ is Dirichlet given $\boldsymbol{\Omega}_j^0$ and the usage modifier variable posteriors are multinomial conditioned on $\boldsymbol{\Omega}_j^k$, the sampling posterior will simply be a Dirichlet distribution:

$$\mathrm{p}(\boldsymbol{\Omega}_j^k \mid \mathbf{u}, \boldsymbol{\Omega}_j^k, \alpha_\Omega) \propto \text{Dirichlet}(\boldsymbol{\Omega}_j^k \mid \alpha_\Omega \boldsymbol{\Omega}_{j1}^0 + c_1^{jk}, \ldots, \alpha_\Omega \boldsymbol{\Omega}_{jV}^0 + c_V^{jk})$$

Here, c_v^{jk} denotes the number of tissues in group k using expression program j with pattern type v.

In step 6, we need to sample from the posterior distributions for the root level usage modifier variables priors, $\boldsymbol{\Omega}_j^0$. Sampling from these posteriors uses an auxiliary variable sampling scheme essentially the same as described in Section A.3 for sampling Hierarchical Dirichlet Process mixture weights. As it turns out, the same sampling method can be used for a finite Dirichlet distribution with only a slight modification. As before, we introduce auxiliary variables $\mathbf{m}$. The conditional distributions for sampling $\mathbf{m}$ and $\boldsymbol{\Omega}_j^0$ are then:

$$\mathrm{p}(\mathsf{m}_{kjv} = m \mid \mathbf{u}, \mathbf{m}_{-kjv}, \boldsymbol{\Omega}_{jv}^0) \propto s(n_{kjv}, m)(\alpha_\Omega \boldsymbol{\Omega}_{jv}^0)^m$$

$$\mathrm{p}(\boldsymbol{\Omega}_j^0 \mid \mathbf{m}) \propto \text{Dirichlet}(\sum_k m_{kj1}, \ldots, \sum_k m_{kjV})$$

Here, $s(\cdot, \cdot)$ denotes a Stirling number of the first kind and n_{kjv} is the number of tissues in group k using expression program j with value v.

We also sample the concentration parameter α_Ω (Step 9) using an auxiliary variable scheme essentially the same as described in section A.3 for sampling Hierarchical Dirichlet Process concentration parameters. We introduce two auxiliary variables $\mathbf{w}$ and $\mathbf{b}$. The update equations are then given by:

$$\mathrm{p}(\mathsf{w}_{kj} \mid \alpha_\Omega) \propto w_{kj}^{\alpha_\Omega}(1 - w_{kj})^{T_k - 1}$$

$$\mathrm{p}(\mathsf{b}_{kj} \mid \alpha_\Omega) \propto \left(\frac{T_k}{\alpha_\Omega}\right)^{b_k j}$$

$$\mathrm{p}(\alpha_\Omega \mid \mathbf{w}, \mathbf{b}) \propto \text{Gamma}(a_1^{\alpha_\Omega} + \sum_{k=1}^{K}\sum_{j=1}^{J}(M_{kj} - b_{kj}), a_2^{\alpha_\Omega} - \sum_{k=1}^{K}\sum_{j=1}^{J}\log w_{kj})$$

Here, T_k is the number of tissues in group k, $a_1^{\alpha_\Omega}$ and $a_2^{\alpha_\Omega}$ are the hyperparameters for the Gamma prior on α_Ω and $M_{kj} = \sum_{v=1}^{V} m_{kjv}$.

We implemented the sampling scheme in Java. Inference was always started with all data assigned to a single expression program. We burned in the sampler for

100,000 iterations, and then collected relevant posterior distribution statistics from 10,000 samples (see Section 1.3). We set the hyperparameters for all concentration parameters to 10^{-8} to produce vague prior distributions. Both hyperparameters for the Gamma prior on λ were set to 1.0, biasing λ toward a unit pseudo-count Dirichlet distribution.

1.3 Consensus tissue groups and recurrent expression programs

We use two methods to summarize the posterior distribution samples: consensus tissue groups (CTGs) and recurrent expression programs (REPs).

CTGs are constructed by first computing the empirical probability that a pair of tissues will be assigned to the same tissue group. The empirical co-grouping probabilities are then used as pair-wise similarity measures in a standard bottom-up agglomerative hierarchical clustering algorithm using complete linkage (e.g., as discussed in [5]). To be precise, let S denote the total number of samples, and $q_t^{(l)}$ the tissue group assignment for tissue t in sample l. The empirical co-grouping probability for tissues t and r is then:

$$\widehat{p}_{tr} = \sum_{l=1}^{S} \mathrm{I}(q_t^{(l)} = q_r^{(l)})/S$$

Here, $I(\cdot)$ is the indicator function.

Clustering is stopped using a pre-defined cut-off c_{tg} to produce the final CTGs. We used a cut-off of $c_{tg} = 0.90$ to produce strongly coherent groups. However, we note that the empirical co-grouping probabilities tend to be either very small or close to 1.0, rendering our results relatively insensitive to the choice of c_{tg}.

REPs consist of sets of tissues and genes that appear together with significant probability in expression programs across multiple samples. After burn-in, we save a predetermined number of samples and then sequentially merge similar programs across samples based on how similar the gene expression probabilities are for programs. Similarity is calculated using the Hellinger distance, a general distance metric for probability measures [3]. To be precise, the Hellinger distance between expression programs j_1 and j_2 is calculated as:

$$D(\boldsymbol{\theta}_{j_1}||\boldsymbol{\theta}_{j_2}) = \sum_{g=1}^{G} \sqrt{\theta_{gj_1}\theta_{gj_2}}$$

Here, G is the total number of genes. Expression programs are merged if the distance between them is less than some threshold c_2 (we used $c_2 = 0.50$).

For tissue t using expression program j in sample s, the tissue usage score $v_{tj}^{(s)}$ is calculated as:

$$v_{tj}^{(s)} = \sum_{i} \theta_{\omega(x_{ti})j} I(z_{ti}^{(s)} = j)$$

Here, $\omega(\cdot)$ is the function mapping expression events to genes, $\boldsymbol{\theta}_j$ is the probability vector for genes in program j, $I(\cdot)$ is the indicator function, and $z_{ti}^{(s)}$ is a variable denoting the assignment of expression event i to an expression program in iteration s. Note that this score will be higher if a tissue uses more genes from the program, regardless of the total number of expression events in the tissue. Further, the score will be higher if a tissue uses genes with large $\boldsymbol{\theta}_j$ values (i.e., higher probabilities of being expressed). Thus, the score reflects how "typical" a tissue's usage of an expression program is. A tissue t is reported as associated with a REP j if its mean usage score $\overline{v}_{tj}$ is greater than some threshold c_1 (we used $c_1 = 0.25$).

The empirical mean expression level $\overline{e}_{gj}$ for gene g in REP j is defined as:

$$\overline{e}_{gj} = \sum_{s=1}^{S} \frac{\sum_{t,i} \mathrm{I}(z_{ti}^{(s)} = j)\theta_{gj}}{S \sum_{t=1}^{T} v_{tj}^{(s)}} \text{ s.t. } \omega(x_{ti}) = g$$

We use 1,000 samples to generate REPs as follows. After burn-in of the MCMC sampler for 100,000 iterations, 10,000 samples are generated, with 1,000 samples saved and 100 iterations between each saved sample discarded. Note that spaced samples from the MCMC sampler better approximate independent samples from the posterior, and can thus result in more accurate results [10]

After all potential REPs are generated, infrequently occurring REPs and genes are filtered for the final output. We filter out REPs that occur in fewer than 50% of samples, and filter out genes with $\overline{e}_{gj}$ scores less than 0.05.

1.4 Generality score

The generality score is the entropy of the normalized distribution of usage of an expression program by all tissues in each group. Because the distribution is normalized, tissue groups that only use an expression program a relatively small amount will have little effect on the score. Thus, a high generality score indicates that an expression program is used relatively evenly across many tissue groups; a low score indicates usage of the program is concentrated among a small number of tissue groups. Note that the generaliry score is computed for each expression program in each MCMC sample, and then averaged across all samples when REPs are constructed.

The algorithm computes the usage $h_{kj}^{(s)}$ for tissue group k of REP j in sample s as:

$$h_{kj}^{(s)} = \sum_{t=1}^{T} v_{tj}^{(s)} \mathrm{I}(q_t^{(s)} = k)$$

Here, $q_t^{(s)}$ is the assignment of tissue t to a tissue group in sample s, and $v_{tj}^{(s)}$ is the tissue usage score described in Section 1.3. The $h_{kj}^{(s)}$ values are then normalized across all tissue groups in the sample, i.e.,:

$$\widehat{h}_{kj}^{(s)} = \frac{h_{kj}^{s}}{\sum_{l=1}^{K} h_{lj}^{(s)}}$$

Here, K is the total number of tissue groups in the sample. The generality score for expression program j in sample s is then computed as:

$$\mathrm{GS}_j^{(s)} = -\sum_{k=1}^{K} \widehat{h}_{kj}^{(s)} \log \widehat{h}_{kj}^{(s)} \tag{1.1}$$

The final generality score for a REP is then simply the mean of generality scores computed in equation 1.1, averaged across all samples in which the relevant expression programs occur.

1.5 Expression data discretization

Expression data input into GeneProgram was first discretized using a mutual information-based greedy agglomerative merging algorithm, essentially as described in Hartemink *et al.* [11].

For completeness, we describe the discretization algorithm here. We begin by initializing the algorithm with sets of expression levels for each tissue. We denote gene i in tissue t by g_{ti}, where there are T tissues. Let $r(g_{ti})$ denote the rank of gene i in tissue t based on the continuous expression value of the gene. To initialize the algorithm, we begin by assigning genes in each tissue t to an ordered set $\Lambda_t^{(0)}$ of N_L discrete expression levels that induce uniform bins on the gene rankings for the tissue. That is, $\Lambda_t^{(0)} = (L_{t1}^{(0)}, \ldots, L_{tN_L}^{(0)})$, where $g_{ti} \in L_{tl}^{(0)}$ iff $l - 1 < r(g_{ti})N_L/G_t \leq l$. Here, G_t is the number of genes in tissue t that are considered expressed (e.g., expression values greater than some threshold).

Each iteration consists of a set of trial merges, in which adjacent levels are merged and a score is computed. For iteration q and for each trial h, the adjacent levels h and $h+1$ are merged, forming a new set of levels with one less element, i.e., $(L_{t1}^{(q-1)}, \ldots, L_{th}^{(q-1)} \bigcup L_{t(h+1)}^{(q-1)}, L_{t(h+2)}^{(q-1)}, \ldots, L_{t(N_L-q)}^{(q-1)})$. Let $\boldsymbol{e}_t^{(qh)}$ denote the discrete vector of expression levels for tissue t for iteration q of the algorithm and trial merge h. That is, $e_{ti}^{(qh)} = l$ iff g_{ti} is in level l for trial merge h and g_{ti} is expressed in the tissue (otherwise, we set $e_{ti}^{(qh)} = 0$). The score for a trial merge h is the mutual information between all pairs of vectors of discretized expression data, i.e., $S_h^q = \sum_{t_1=1}^{T-1} \sum_{t_2>t_1} \mathrm{MI}(\boldsymbol{e}_{t_1}^{(qh)}, \boldsymbol{e}_{t_2}^{(qh)})$. At each iteration, the single merge operation that produces the highest score is retained. Note that because the algorithm is greedy, its run-time is $O(N_L^2 T^2)$.

1.6 Synthetic data

In creating synthetic data, we sought to simulate important features of real microarray data profiling mammalian tissues. Thus, we assumed noisy data in which there were several distinct populations of related tissues using different sets of co-expressed genes.

We generated four gene sets used by 40 tissues divided equally among four tissue populations. Each gene set contained 40 genes with varying mRNA levels; gene sets

gene set no.	tissue pop. 1	tissue pop. 2	tissue pop. 3	tissue pop. 4
1	30	25	3	3
2	3	30	25	3
3	5	3	37	20
4	3	3	20	20

Table 1.2: Tissue population means for synthetic data. Each tissue population was associated with a mean vector of the numbers of genes to be used from each gene set. For a tissue from a population, the number of genes to be used from a gene set was sampled from a Poisson distribution using the population mean.

three and four overlapped in 10 genes. The simulated underlying mRNA level m_{ij} for gene i in gene set j was generated as $m_{ij} \sim \text{round}(1000 * \text{Gamma}(3, 2))$.

Each tissue population k was associated with a mean vector $\boldsymbol{N}_k$ of the numbers of genes to be used from each gene set (see Table 1.2 for the mean vectors used to generate the simulated data). For a tissue t from population k, the number of genes to be used from gene set j was sampled from a Poisson distribution with parameter N_{kj}.

Genes were picked to be expressed from each set used by the tissue. Genes were picked without replacement such that the probability of picking gene i from gene set j for tissue t when l genes had already been picked was:

$$p_{ti}^{(l)} = \frac{\log m_{ij}}{\sum_{k \notin G_{tj}^{(l-1)}} \log m_{kj}}$$

Where genes were picked sequentially and $G_{tj}^{(l-1)}$ denotes the collection of the first $l-1$ genes picked from gene set j for tissue t. The probability is zero if the gene had already been picked. Finally, the observed expression value e_{it} for gene i in tissue t was generated as:

$$e_{it} = a_{it} I_{tij} m_{ij} + b_{it}$$

Here, I_{tij} is an indicator denoting whether gene i from gene set j was picked by tissue t, and a_{it} and b_{it} are multiplicative and additive noise respectively. Noise was generated with $a_{it} \sim \text{lognormal}(0, 0.1)$ and $b_{it} \sim \text{lognormal}(\log(200), 1)$. The mean and scale of noise were chosen to approximate Affymetrix microarray data (see [18]).

We note that our scheme for simulating data does not simply recapitulate the assumptions present in the GeneProgram model (e.g., it does not assume discrete and independent "units" of expression signal and it introduces microarray-like noise).

APPENDIX A

Dirichlet Processes Overview

A.1 Introduction

The task of assigning data to clusters is a classic problem in machine learning and statistics. A common approach to this problem is to construct a model in which data is generated from a mixture of probability distributions.

In finite mixture models, data is assumed to arise from a mixture with a predetermined number of components [14]. The difficulty with such models is that the appropriate number of mixture components is not known *a priori* for many modeling applications. This issue is generally addressed by constructing a series of models with different numbers of components, and evaluating each model using some quality score [14].

An alternate, fully Bayesian approach is to build an *infinite* mixture model, in which the number of mixture components is potentially unlimited, and is itself a random variable that is part of the overall model. Obviously, only a finite number of mixture components can have data assigned to them. However, we still imagine the data as arising from an infinite number of components; as more data is collected, more components may be used to model the data more accurately. Thus, the infinite mixture model is a nonparametric model, in the sense that the number of model parameters grows with the amount of data. The challenge with such a model is how to place an appropriate prior on the infinite number of mixture component parameters and weights.

The Dirichlet Process (DP), a type of stochastic process first introduced in the 1960's [9] and originally of mostly theoretical interest [7, 8], has recently become an important modeling tool as a prior distribution for infinite mixture models. In this appendix, we will introduce the main concepts of DPs necessary to understand the GeneProgram model. In this regard, we will focus on a constructive definition of DPs in the context of priors for infinite mixture models. This development, which avoids

measure theory, closely parallels that presented by [16] and [19].

A recent extension to the standard DP model is the Hierarchical Dirichlet Process (HDP), in which dependencies are specified among a set of DPs by arranging them in a tree structure [21]. HDPs are useful as priors for hierarchical mixture models, in which data is arranged into populations that preferentially share the usage of mixture components. Here, we will discuss the original HDP formulation by Teh *et al.* in the context of infinite mixture models.

The use of DPs for real-world applications is predicated on practical inference methods. A great advance in this regard has been the development of efficient Markov Chain Monte Carlo (MCMC) methods for approximate inference for infinite mixture models using DP priors [20, 17, 19]. Although other approximate inference methods have been developed [15, 4, 13], MCMC remains the most widely used and versatile method. In particular, efficient MCMC schemes have been developed for HDP models [21], and can be readily extended for the GeneProgram model. Thus, our discussion of DP inference in this appendix will be restricted to MCMC methods.

The remainder of this appendix is organized as follows. First, we describe how Dirichlet Processes arise as priors in terms of the infinite limit of mixture models. Next, we describe the extension of DPs to HDPs. Finally, we describe basic MCMC sampling schemes for DPs and HDPs.

A.2 Probability models

A.2.1 Bayesian finite mixture models

We begin by defining a typical Bayesian finite mixture model, which we will subsequently extend to the infinite case. Figure A-1 depicts the model using standard graphical model notation with plates. The model consists of J mixture components, where each component j has associated with it a mixture weight denoted π_j and a parameter denoted θ_j. Assume we have N data points denoted x_i, where $1 \leq i \leq N$. Each data point is assigned to a mixture component via an indicator variable z_i, i.e., the probability that data point i is assigned to component j is $\mathrm{p}(\mathsf{z}_i = j \mid \boldsymbol{\pi}) = \pi_j$ or $\mathsf{z}_i \mid \boldsymbol{\pi} \sim \text{Multinomial}(\cdot \mid \boldsymbol{\pi})$. The conditional likelihood for each data point may then be written as:

$$\mathrm{p}(\mathsf{x}_i \mid \mathsf{z}_i = j, \boldsymbol{\theta}) = F(\mathsf{x}_i \mid \theta_j)$$

Here, $F(\cdot \mid \cdot)$ is a probability density function parameterized by $\boldsymbol{\theta}$.

To complete the model, we need to define prior distributions over the parameters. We will assume that the component parameters are drawn i.i.d. from some base distribution H, i.e., $\theta_j \sim H(\cdot)$. We also need to specify a prior distribution for the weight parameters. As is typical for Bayesian mixture models, we will assume a symmetric Dirichlet prior on the mixture weights, i.e., $\boldsymbol{\pi} \mid J, \alpha \sim \text{Dirichlet}(\cdot \mid \alpha/J)$. One consequence of using a symmetric prior is that it is not sensitive to the order of the component parameters. Note that the Dirichlet prior is conjugate to the multinomially distributed weights, so that the posterior is also a Dirichlet distribution.

To summarize, our J-dimensional mixture model is defined as:

$$\boldsymbol{\pi} \mid \alpha, J \sim \text{Dirichlet}(\cdot \mid \alpha/J)$$

$$\theta_j \mid H \sim H(\cdot)$$
$$\mathsf{z}_i \mid \boldsymbol{\pi} \sim \text{Multinomial}(\cdot \mid \boldsymbol{\pi})$$
$$\mathsf{x}_i \mid \mathsf{z}_i = j, \boldsymbol{\theta} \sim F(\cdot \mid \theta_j)$$

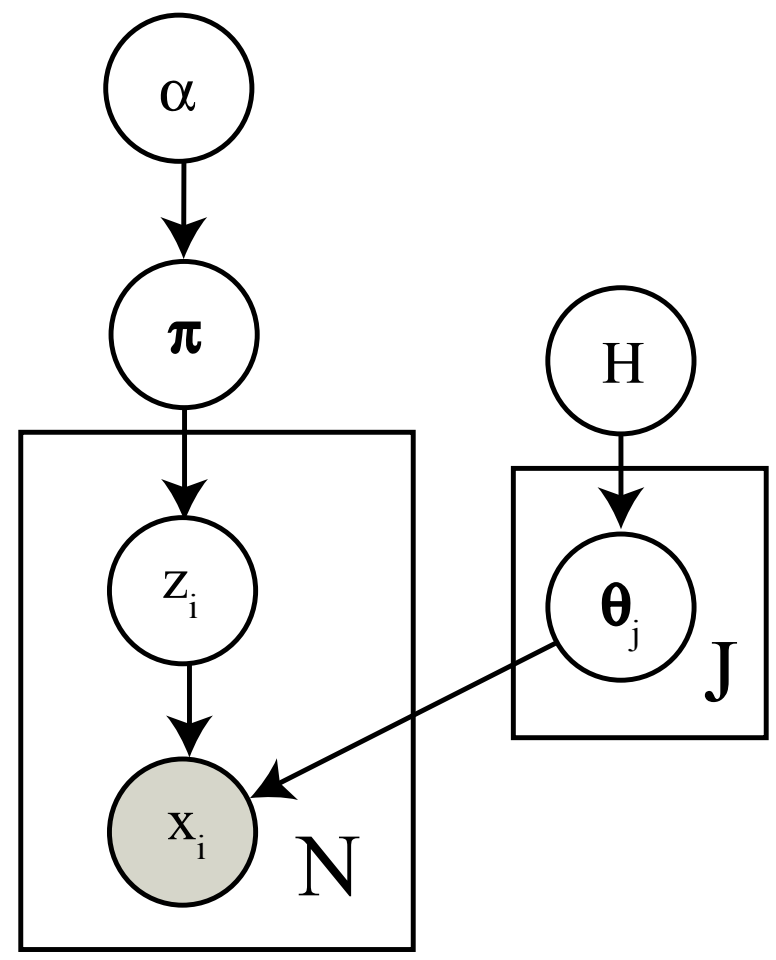

Figure A-1: A graphical model depiction of a finite mixture model with J mixture components and N data items. Circles represent variables, and arrows denote dependencies among variables. Vectors are depicted with bold type, and observed variables are shown inside shaded circles. Rectangles represent plates, or repeated sub-structures in the model.

In mixture models, we are primarily interested in knowing which component each data point i has been assigned to—the weights $\boldsymbol{\pi}$ are to some extent "nuisance" parameters. It is possible to derive closed form expressions for the data point assignment variable posterior distributions with the mixture weights integrated out. These posterior distributions will be particularly useful in the extension to the infinite mixture model. Note that although the assignment variables $\mathbf{z}$ are conditionally independent given the weights, they become dependent if we integrate out the weights (i.e., the probability of assigning a data point to a particular component depends on the assignments of all other data points). As it turns out, the probability of assigning data point i to some component j given assignments of all other data points can be written as a simple closed form expression (see [19]):

$$\text{p}(\mathsf{z}_i = j \mid \mathbf{z}_{-i}, \alpha, J) = \int \text{p}(\mathsf{z}_i = j \mid \boldsymbol{\pi})\text{p}(\boldsymbol{\pi} \mid \mathbf{z}_{-i}, \alpha, J) d\boldsymbol{\pi}$$

$$\text{p}(\boldsymbol{\pi} \mid \mathbf{z}_{-i}, \alpha, J) \propto \text{p}(\mathbf{z}_{-i} \mid \boldsymbol{\pi})\text{p}(\boldsymbol{\pi} \mid \alpha, J)$$

$$\Rightarrow \text{p}(\mathsf{z}_i = j \mid \mathbf{z}_{-i}, \alpha, J) \propto \int \text{p}(\mathsf{z}_i = j \mid \boldsymbol{\pi})\text{p}(\mathbf{z}_{-i} \mid \boldsymbol{\pi})\text{p}(\boldsymbol{\pi} \mid \alpha, J) d\boldsymbol{\pi}$$

$$\Rightarrow \mathrm{p}(\mathsf{z}_i = j \mid \mathbf{z}_{-i}, \alpha, J) \propto \frac{n_j^{-i} + \alpha/J}{N - 1 + \alpha} \tag{A.1}$$

Here, $\mathbf{z}_{-i}$ denotes the assignments of all data excluding data point i, and n_j^{-i} denotes the number of data points assigned to component j excluding data point i. The second line of the derivation follows simply from Bayes' theorem. The final line of the derivation follows from conjugacy between the Dirichlet prior on the weights and the multinomial distribution on the assignment variables. Thus, the density function under the integral is that of a non-symmetrical Dirichlet distribution, allowing us to derive the final closed form expression.

A.2.2 Infinite mixture models and Dirichlet Processes

In this subsection we show how the Dirichlet Process arises as a prior for infinite mixture models.

Figure A-2 depicts an infinite mixture model using standard graphical model notation with plates. As can be seen from the figure, the model is almost structurally identical to the finite version. The distinguishing feature is that the weight and parameter vectors are now infinite dimensional.

The challenge with this model is then to define an appropriate prior for the infinite dimensional parameters and weights. As with any mixture model, the infinite dimensional weights must sum to one. A probability distribution that generates such weights is the *stick-breaking* distribution, denoted Stick(α), where α is a scaling or concentration parameter (discussed in more detail below). This distribution is defined constructively. Intuitively, we imagine starting with a stick of unit length and breaking it at a random point. We retain one of the pieces, and break the second piece again at a random point. This process is repeated infinitely, producing a set of random weights that sum to one with probability one [20]. To be more precise, the jth weight π_j is constructed as:

$$\pi'_j \mid \alpha \sim \mathrm{Beta}(1, \alpha)$$

$$\pi_j = \pi'_j \prod_{l=1}^{j-1} (1 - \pi'_l)$$

The infinite mixture model can be constructed using the stick-breaking distribution as a prior on the mixture weights and the base distribution H as a prior on the component parameters. This can be summarized as:

$$\boldsymbol{\pi} \mid \alpha \sim \mathrm{Stick}(\alpha)$$

$$\theta_j \mid H \sim H(\cdot)$$

$$\mathsf{z}_i \mid \boldsymbol{\pi} \sim \mathrm{Multinomial}(\cdot \mid \boldsymbol{\pi})$$

$$\mathsf{x}_i \mid \mathsf{z}_i = j, \boldsymbol{\theta} \sim F(\cdot \mid \theta_j)$$

Note that this construction produces a vector $\boldsymbol{\pi}$ with a countably infinite number

of dimensions, whose components all sum to one, and H is sampled independently a countably infinite number of times to generate the mixture component parameter values.

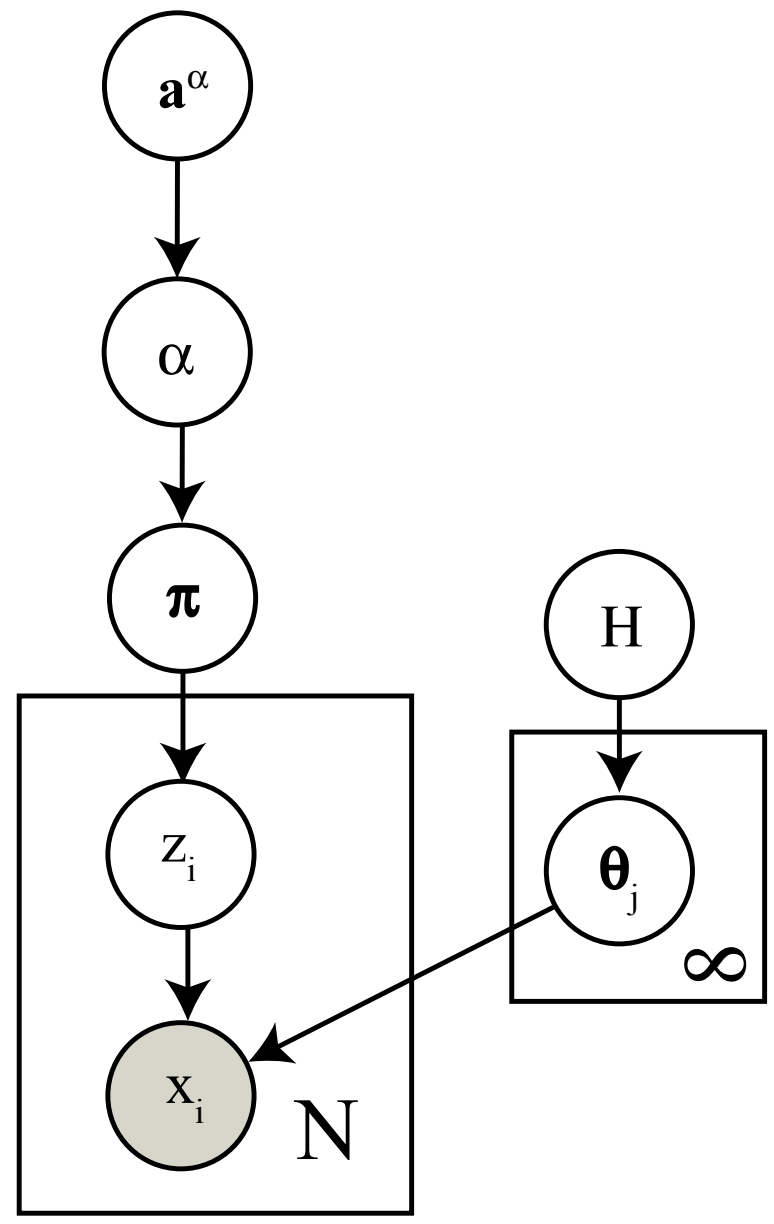

Figure A-2: A graphical model depiction of the infinite mixture model. Circles represent variables, and arrows denote dependencies among variables. Vectors are depicted with bold type, and observed variables are shown inside shaded circles. Rectangles represent plates, or repeated sub-structures in the model.

To establish the connection between Dirichet Processes and the model described above, we consider the distribution over all possible component parameter values for the infinite mixture model. This distribution will be non-zero at a countably infinite number of values. Formally, we denote this distribution by G and can write it as:

$$G(\psi) = \sum_{j=1}^{\infty} \pi_j \delta(\psi - \theta_j)$$

Here, ψ is an arbitrary parameter value, and $\delta(\cdot)$ is the standard delta-function, which is non-zero only when its argument is zero.

Each distribution G thus constructed can be viewed as a sample from a stochastic process, which can in fact be proven to be the Dirichlet Process (see [12] and [20]). In general, we will characterize a Dirichlet Process by a scalar parameter α, called the concentration parameter, and a base distribution H. A sample from a Dirichlet Process, which we denote $G\,|\alpha, H \sim \mathrm{DP}(\alpha, H)$, is thus a distribution that is non-zero over a countably infinite number of values (with probability one). As we have seen, each sample effectively parameterizes an infinite dimensional mixture model.

The concentration parameter α affects the expected number of mixture components containing data items when the DP is used as a prior for the infinite mixture

model. As shown in [2], the expected number of non-empty mixture components J depends only on α and the number of data points N:

$$E[J \mid \alpha, N] = \alpha \sum_{l=J-1}^{N} \frac{1}{\alpha + l - 1} \approx \alpha \ln\left(\frac{N+\alpha}{\alpha}\right)$$

Thus, we see that the number of non-empty components grows approximately as the logarithm of the size of the data set. Further, we see that the number of components grows as the concentration parameter α increases.

To make our model fully Bayesian, we would like to treat the concentration parameter α as a random variable and place a prior on it. The Gamma distribution is commonly used as a prior for α, in part because efficient inference is possible with this prior, and also because appropriate parameter choices result in a relatively uninformative prior [16]. Thus, we place a Gamma prior on α with hyperparameters $\boldsymbol{a}^\alpha$, i.e., $\alpha \mid \boldsymbol{a}^\alpha \sim \text{Gamma}(a_1^\alpha, a_2^\alpha)$.

A.2.3 Hierarchical Dirichlet Process models

In this section, we describe the Hierarchical Dirichlet Process (HDP) models introduced by Teh *et al.* [21]. As in the previous section on DPs, we will present HDPs in terms of priors for infinite mixture models. We will describe only a two-level hierarchical model for clarity; additional levels are simply added by applying the model construction process recursively.

Figure A-3 depicts a basic HDP using standard graphical model notation with plates. In HDP models, we assume that data is divided into T subsets, each consisting of N_t data points denoted x_{ti}, where $1 \leq t \leq T$ and $1 \leq i \leq N_t$. Each such data set division is modeled by an infinite mixture model with weights $\boldsymbol{\pi}_t$ and component assignment variables z_{ti}. These infinite mixture models are not independent; the mixtures share component parameters $\boldsymbol{\theta}$ and a common Dirichlet Process prior.

The dependencies among the infinite mixture models can be understood in terms of a construction using the stick-breaking distribution. Beginning at the top of the model, we imagine drawing a sample G from a Dirichlet Process, i.e., $G \mid \alpha_0, H \sim \text{DP}(\alpha_0, H)$. Recall that we can write this sample as:

$$G(\psi) = \sum_{j=1}^{\infty} \beta_j^0 \delta(\psi - \theta_j)$$

Here, θ_j are drawn i.i.d. from the base distribution H, and $\boldsymbol{\beta}^0 \mid \alpha_0 \sim \text{Stick}(\alpha_0)$.

We next form a second DP using the sample G itself as a base distribution, i.e., we construct $\text{DP}(\alpha_1, G)$. We then generate i.i.d. samples from this DP for each of the T sub-models, i.e., $G_t \mid \alpha_1, G \sim \text{DP}(\alpha_1, G)$. Each sample can be written as:

$$G_t(\psi) = \sum_{j=1}^{\infty} \pi_{tj} \delta(\psi - \theta_j)$$

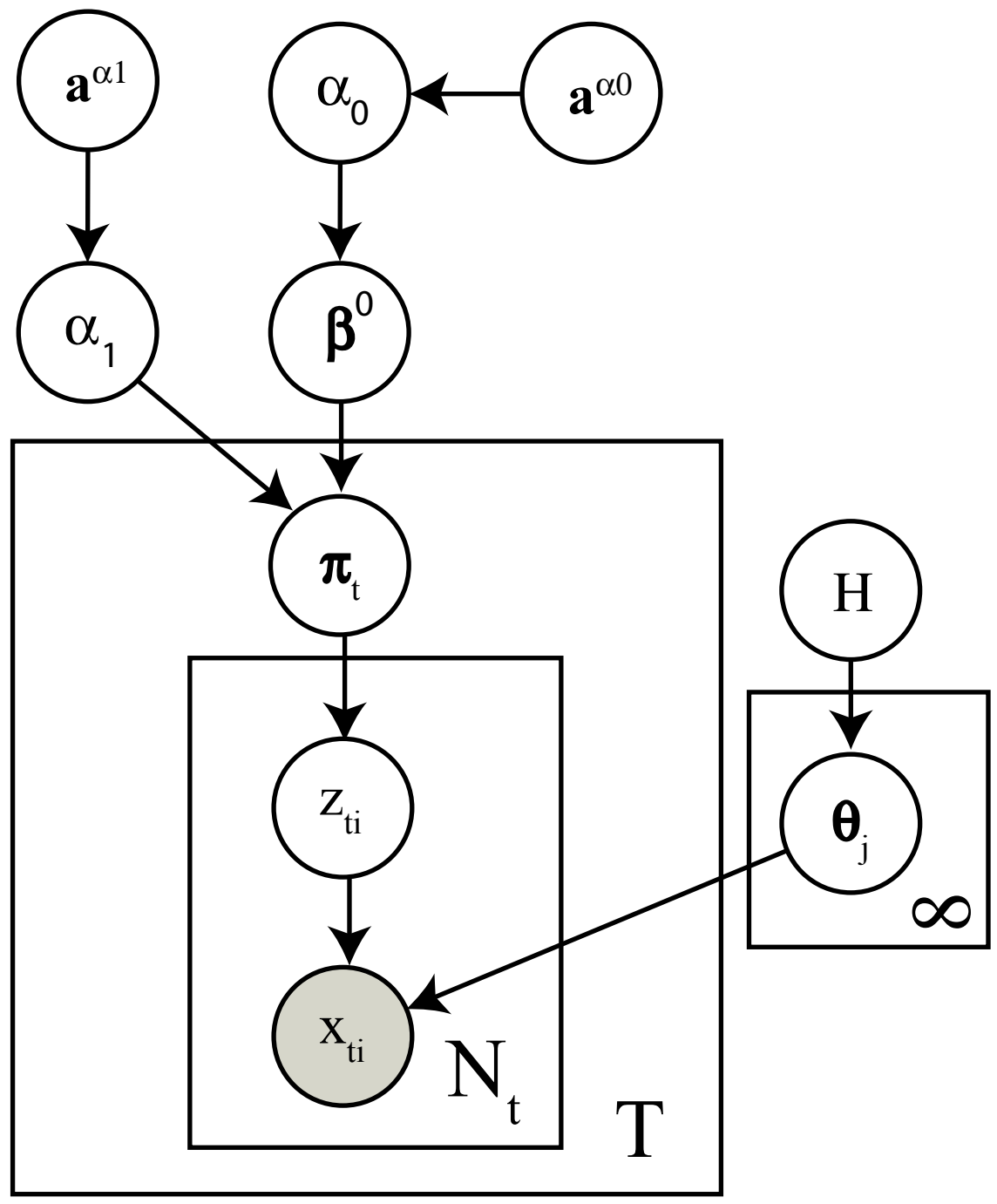

Figure A-3: A graphical model depiction of the Hierarchical Dirichlet process represented as an infinite mixture model. Circles represent variables, and arrows denote dependencies among variables. Vectors are depicted with bold type, and observed variables are shown inside shaded circles. Rectangles represent plates, or repeated sub-structures in the model.

Notice that these distributions must necessarily be non-zero only at the same points θ_j as G is. We have now constructed a set of T dependent infinite mixture models, where each model has separate (but dependent) weights $\boldsymbol{\pi}_t$ and shared component parameters $\boldsymbol{\theta}$.

It can be shown that the weights $\boldsymbol{\pi}_t$ can be constructed via a stick-breaking process using the top-level weights $\boldsymbol{\beta}^0$ (see [21]):

$$\pi'_{tj} \sim \text{Beta}\left(\alpha_1 \beta^0_j, \alpha_1 \left(1 - \sum_{l=1}^{j} \beta^0_l\right)\right)$$

$$\pi_{tj} = \pi'_{tj} \prod_{l=1}^{j-1} (1 - \pi'_{tl})$$

A.3 Markov Chain Monte Carlo inference for infinite mixture models

A.3.1 Single level infinite mixture models

Markov Chain Monte Carlo (MCMC) algorithms are general tools for approximating posterior distributions of models. With these methods, one alternately samples from the distributions for subsets of variables conditioned on the remaining variables. Given some mild constraints on the model distributions, the approximation converges to the true posterior distribution in the large sample limit [10]. The utility of MCMC methods hinges on the ability to sample from a set of conditional distributions more efficiently than sampling from the full posterior.

In the case of infinite mixture models using a DP prior, sampling can be made efficient by exploiting a "trick" that requires tracking of only a finite number of non-empty mixture components and the data points already assigned to them. Figure A-4 presents the overall MCMC sampling scheme for single level infinite mixture models.

Repeat for all data items $i = 1 \ldots N$:
- Sample z_i, the assignment of the data item to a mixture component, from its posterior, i.e., $\mathrm{p}(\mathsf{z}_i \mid \mathbf{z}_{-i}, \alpha, \boldsymbol{\theta})$
  - If the data item has been assigned to a new component, sample a new mixture component parameter θ_* from its posterior

Repeat for all non-empty mixture components $j = 1 \ldots J$:
- Sample the component parameter θ_j from its posterior

Sample the DP concentration parameter α from its posterior

Figure A-4: One iteration of the basic MCMC sampling scheme for an infinite mixture model using a Dirichlet Process prior.

The key MCMC sampling step for Dirichlet Processes involves picking assignments of data points to mixture components. We sample the assignment of a data point i conditioned on the other variables from the distribution given by:

$$\mathrm{p}(\mathsf{z}_i \mid \mathbf{z}_{-i}, \alpha, \boldsymbol{\theta}, \mathbf{x}) \propto \mathrm{p}(\mathsf{z}_i \mid \mathbf{z}_{-i}, \alpha)\mathrm{p}(\mathbf{x} \mid \mathbf{z}, \boldsymbol{\theta}) \tag{A.2}$$

The proportionality simply follows from Bayes' theorem. Recall from equation A.1 that for finite mixture models, we can write $\mathrm{p}(\mathsf{z}_i = j \mid \mathbf{z}_{-i}, \alpha, J)$ in closed form:

$$\mathrm{p}(\mathsf{z}_i = j \mid \mathbf{z}_{-i}, \alpha, J) \propto \frac{n_j^{-i} + \alpha/J}{N - 1 + \alpha}$$

For the case of infinite mixture models, and in which $n_j^{-i} > 0$ (i.e., the jth component of the mixture is non-empty), it can be proven that this distribution converges to

(see [19]):

$$\mathrm{p}(\mathsf{z}_i = j \mid \mathbf{z}_{-i}, \alpha) \propto \frac{n_j^{-i}}{N - 1 + \alpha} \tag{A.3}$$

For infinite mixture models, we must consider the probability that a data point does not belong to one of the mixture components containing other data points. That is, we will need to calculate $\mathrm{p}(\mathsf{z}_i \neq \mathsf{z}_l, \forall\, l \neq i \mid \mathbf{z}_{-i}, \alpha)$. It can be proven that this probability is given by (see [19]):

$$\mathrm{p}(\mathsf{z}_i \neq \mathsf{z}_l, \forall\, l \neq i \mid \mathbf{z}_{-i}, \alpha) \propto \frac{\alpha}{N - 1 + \alpha} \tag{A.4}$$

We can thus combine equations A.2, A.3 and A.4 to obtain the posterior distributions for the assignment variables:

$$\mathrm{p}(\mathsf{z}_i = j \mid \mathbf{z}_{-i}, \alpha, \boldsymbol{\theta}, \mathbf{x}) \propto \frac{n_j^{-i}}{N - 1 + \alpha} \mathrm{p}(\mathsf{x}_i \mid \theta_j) \quad \text{for } n_j^{-i} > 0 \tag{A.5}$$

$$\mathrm{p}(\mathsf{z}_i \neq \mathsf{z}_l, \forall\, l \neq i \mid \mathbf{z}_{-i}, \alpha, \boldsymbol{\theta}, \mathbf{x}) \propto \frac{\alpha}{N - 1 + \alpha} \int F(\mathsf{x}_i \mid \psi) H(\psi) d\psi \tag{A.6}$$

Thus, for each iteration, we sample the mixture component assignments for all data points using equations A.5 and A.6. For the first J components already containing data items, we use equation A.5 to compute the assignment probability. We use equation A.6 to compute the probability of assigning the data point to a new mixture component. Notice that in equation A.6, we integrate over the mixture component parameter, as any component parameter is possible for a new component. Sampling is most efficient when $F(\cdot)$ and $H(\cdot)$ are conjugate. However, in cases of non-conjugacy of these distributions, Monte Carlo methods may be used [17, 19].

We also need to sample from the posterior for the concentration parameter α. It can be shown that the conditional distribution for α is given by (see [16]):

$$\mathrm{p}(\alpha \mid J, N, \boldsymbol{a}^{\alpha}) \propto \alpha^{a_1^{\alpha} + J - 1} e^{-a_2^{\alpha} \alpha} \mathrm{B}(\alpha, N)$$

Here, $\mathrm{B}(\cdot, \cdot)$ is the standard Beta function defined as:

$$\mathrm{B}(u, v) = \frac{\Gamma(u)\Gamma(v)}{\Gamma(u + v)} = \int_0^1 \eta^{u-1} (1 - \eta)^{v-1} d\eta$$

Escobar and West describe an efficient sampling scheme for α [6]. They noted that $\mathrm{p}(\alpha \mid J, N, \boldsymbol{a}^{\alpha})$ can be written as a marginalization over an auxiliary variable η:

$$\mathrm{p}(\alpha \mid J, N, \boldsymbol{a}^{\alpha}) \propto \int_0^1 \mathrm{p}(\alpha, \eta \mid J, N) d\eta$$

$$\mathrm{p}(\alpha, \eta \mid J, N) \propto \alpha^{a_1^{\alpha} + J - 1} e^{-a_2^{\alpha} \alpha} \eta^{\alpha - 1} (1 - \eta)^{N-1}$$

From the joint distribution, we can see that:

$$p(\alpha \mid \eta, J, N, \boldsymbol{a}^{\alpha}) \propto \text{Gamma}(\alpha \mid a_1^{\alpha} + J - 1, a_2^{\alpha} - \ln \eta)$$

$$p(\eta \mid \alpha, J, N) \propto \text{Beta}(\eta \mid \alpha, N)$$

Thus, by sampling from the above two conditional distributions, we can sample from the posterior for α to update the concentration parameter during the MCMC sampling iterations.

A.3.2 Hierarchical Dirichlet Process models

Teh *et al.* described an MCMC method for HDP infinite mixture models that uses auxiliary variables to make sampling from the conditional distributions efficient [21]. Figure A-5 provides an overview of the sampling scheme.

Repeat for all data subsets $t = 1 \ldots T$ and data items $i = 1 \ldots N$:
  Sample z_{ti}, the assignment of data item i from subset t to a mixture component, from its posterior, i.e., $p(\mathsf{z}_{ti} \mid \mathbf{z}_{-i}, \boldsymbol{\beta}^0, \boldsymbol{\theta}, \mathbf{x}, \alpha_1)$
    If the data item has been assigned to a new component, sample a new top-level mixture weight β_*^0 from the stick-breaking distribution and a new mixture component parameter θ_* from its posterior

Repeat for all non-empty mixture components $j = 1 \ldots J$:
  Sample the component parameter θ_j from its posterior

Sample the top-level mixture weights $\boldsymbol{\beta}^0$ from their posterior

Sample the concentration parameters α_0 and α_1 from their posteriors

Figure A-5: One iteration of the basic MCMC sampling scheme for the Hierarchical Dirichlet Process mixture model with two levels.

The first task is to sample the data point assignment variables, $\mathbf{z}$. The method for this is similar to that used for ordinary Dirichlet Process mixture models. We begin by considering a finite mixture model of dimension J and integrating out the individual mixture weights $\boldsymbol{\pi}_t$ to obtain the conditional probability of $\mathbf{z}$ given $\boldsymbol{\beta}^0$:

$$p(\mathbf{z} \mid \boldsymbol{\beta}^0, \alpha_1) = \prod_{t=1}^{T} \frac{\Gamma(\alpha_1)}{\Gamma(\alpha_1 + N_t)} \prod_{j=1}^{J} \frac{\Gamma(\alpha_1 \beta_j^0 + n_{tj})}{\Gamma(\alpha_1 \beta_j^0)} \tag{A.7}$$

Here, N_t denotes the number of data items in subset t, and n_{tj} represents the number of data items from subset t assigned to mixture component j. It can be shown that in the limit of an infinite mixture model, the conditional probability has a particularly simple form:

$$p(\mathsf{z}_{ti} = j \mid \mathbf{z}_{-i}, \boldsymbol{\beta}^0, \alpha_1) \propto \alpha_1 \beta_j^0 + n_{tj}^{-i}$$

By combining this with the conditional likelihood for data points, $F(\cdot \mid \cdot)$, we obtain the posterior distribution for assigning data points to mixture components:

$$\mathrm{p}(\mathsf{z}_{ti} = j \mid \mathbf{z}_{-i}, \boldsymbol{\beta}^0, \boldsymbol{\theta}, \mathbf{x}, \alpha_1) \propto (\alpha_1 \beta_j^0 + n_{tj}^{-i}) F(\mathsf{x}_{ti} \mid \theta_j) \quad \text{(A.8)}$$

This equation holds if j is a non-empty component. The posterior distribution for assigning a data point to a new component is given by:

$$\mathrm{p}(\mathsf{z}_{ti} \neq \mathsf{z}_{tl} \; \forall \, t, l \neq i \mid \mathbf{z}_{-i}, \boldsymbol{\beta}^0, \boldsymbol{\theta}, \mathbf{x}, \alpha_1) \propto (\alpha_1 \beta_*^0) \int F(\mathsf{x}_{ti} \mid \psi) H(\psi) d\psi \quad \text{(A.9)}$$

Here, we define $\beta_*^0 = 1 - \sum_{l=1}^{J} \beta_l^0$, where there are J components with data points assigned to them. As with ordinary DPs, Monte Carlo methods may be used if $F(\cdot \mid \cdot)$ and $H(\cdot)$ are non-conjugate distributions.

So, to sample the data point assignments we use equations A.8 and A.9. If a data point is assigned to a new component, we must also generate a new weight β_{J+1}^0 using the stick-breaking distribution, i.e., we sample $b \sim \mathrm{Beta}(1, \alpha_0)$ and set $\beta_{J+1}^0 \leftarrow b\beta_*^0$.

To sample from the model posterior, we also must sample the top-level weights $\boldsymbol{\beta}^0$. The method for this relies on a "trick" using auxiliary variables. For the derivation, we need to use a general property of ratios of Gamma functions given by:

$$\frac{\Gamma(n+a)}{\Gamma(a)} = \sum_{m=0}^{n} s(n,m) a^m \quad \text{(A.10)}$$

Here, n and a are natural numbers. In equation A.10, the ratio of Gamma functions has been expanded into a polynomial with a coefficient $s(n,m)$ for each term. These coefficients are called unsigned Stirling numbers of the first kind, which count the permutations of n objects having m permutation cycles (see [1]). By definition, $s(0,0) = 1$, $s(n,0) = 0$, $s(n,n) = 1$ and $s(n,m) = 0$ for $m > n$. Additional coefficients are then computed recursively using the equation $s(n+1,m) = s(n,m-1) + ns(n,m)$.

Note that the $\boldsymbol{\beta}^0$ weights in the conditional probability $\mathrm{p}(\mathbf{z} \mid \boldsymbol{\beta}^0)$ in equation A.7 occur as arguments of ratios of Gamma functions. These ratios can be expanded to yield polynomials in the $\boldsymbol{\beta}^0$ weights:

$$\frac{\Gamma(\alpha_1 \beta_j^0 + n_{tj})}{\Gamma(\alpha_1 \beta_j^0)} = \sum_{m_{tj}=0}^{n_{tj}} s(n_{tj}, m_{tj}) (\alpha_1 \beta_j^0)^{m_{tj}} \quad \text{(A.11)}$$

An efficient sampling method can be derived by introducing $\mathbf{m}$ as auxiliary variables. The conditional distributions for sampling $\mathbf{m}$ and $\boldsymbol{\beta}^0$ can be shown to be:

$$\mathrm{p}(\mathsf{m}_{tj} = m \mid \mathbf{z}, \mathbf{m}_{-tj}, \boldsymbol{\beta}^0) \propto s(n_{tj}, m) (\alpha_1 \beta_j^0)^m \quad \text{(A.12)}$$

$$\mathrm{p}(\boldsymbol{\beta}^0 \mid \mathbf{z}, \mathbf{m}) \propto (\beta_*^0)^{\alpha_0 - 1} \prod_{j=1}^{J} \beta_j^{\sum_t m_{tj} - 1} \propto \mathrm{Dirichlet}(\sum_t m_{t1}, \ldots, \sum_t m_{tJ}, \alpha_0) \quad \text{(A.13)}$$

Finally, we need to sample the concentration parameters α_0 and α_1 for the HDP. As with the regular DP model, we will assume Gamma priors on the concentration parameters.

For α_0, it can be shown that:

$$\mathrm{p}(\mathsf{J} = J \mid \alpha_0, \mathbf{m}) \propto s(M, J)\alpha_0^J \frac{\Gamma(\alpha_0)}{\Gamma(\alpha_0 + M)}$$

Here, $M = \sum_t \sum_j m_{tj}$ and J is the number of non-empty mixture components. Combining the above equation with the prior for α_0 yields the conditional probability for α_0, which can be sampled using the same method as described for sampling concentration parameters for regular DPs.

Sampling α_1 requires the introduction of two additional auxiliary variables $\mathbf{w}$ and $\mathbf{b}$. The following update equations can then be derived:

$$\mathrm{p}(\mathsf{w}_t \mid \alpha_1) \propto w_t^{\alpha_1}(1 - w_t)^{N_t - 1}$$

$$\mathrm{p}(\mathsf{b}_t \mid \alpha_1) \propto \left(\frac{N_t}{\alpha_1}\right)^{b_t}$$

$$\mathrm{p}(\alpha_1 \mid \mathbf{w}, \mathbf{b}) \propto \mathrm{Gamma}(a_1^{\alpha_1} + \sum_{t=1}^{T}(M_t - b_t), a_2^{\alpha_1} - \sum_{t=1}^{T} \log w_t)$$

Here, $a_1^{\alpha_1}$ and $a_2^{\alpha_1}$ are the hyperparameters for the Gamma prior on α_1 and $M_t = \sum_{j=1}^{J} m_{tj}$.

Bibliography

[1] M Abramowitz and I. A. Stegun. *Handbook of Mathematical Functions with Formulas, Graphs, and Mathematical Tables*. Dover, 1972.

[2] C. Antoniak. Mixtures of Dirichlet processes with applications to Bayesian non-parametric problems. *Annals of Statistics*, 2(6):1152–1174, 1974.

[3] A. Basu, I. R. Harris, and S. Basu. Minimum distance estimation: The approach using density-based distances. In G. S. Maddala and C. R. Rao, editors, *Handbook of Statistics*, volume 15, pages 21–48. North-Holland, 1997.

[4] David M. Blei and Michael I. Jordan. Variational inference for Dirichlet process mixtures. *Journal of Bayesian Analysis*, 1:121–144, 2005.

[5] M. B. Eisen, P. T. Spellman, P. O. Brown, and D. Botstein. Cluster analysis and display of genome-wide expression patterns. *Proc Natl Acad Sci U S A*, 95(25):14863–8, Dec 8 1998.

[6] Michael D. Escobar and Milke West. Bayesian density estimation and inference using mixtures. *Journal of the American Statistical Association*, 90(430):577–588, 1995.

[7] T.S. Ferguson. A bayesian analysis of some nonparametric problems. *Annals of Statistics*, 1:209–230, 1973.

[8] T.S. Ferguson. Prior distributions on spaces of probability measures. *Annals of Statistics*, 2:615–629, 1974.

[9] D.A. Freedman. On the asymptotic behavior of Bayes estimates in the discrete case. *Annals of Mathematical Statistics*, 34:1386–1403, 1963.

[10] Andrew Gelman, John B. Carlin, Hal S. Stern, and Donald B. Rubin. *Finite Mixture Models*. Chapman & Hall, 2004.

[11] Alexander J. Hartemink, David K. Gifford, Tommi S. Jaakkola, and Richard A. Young. Using graphical models and genomic expression data to statistically validate models of genetic regulatory networks, January 2001.

[12] H. Ishwaran and L. James. Gibbs sampling methods for stick-breaking priors. *Journal of the American Statistical Association*, 96(453):161–173, 2001.

[13] Kenichi Kurihara, Max Welling, and Nikos Vlassis. Accelerated variational Dirichlet process mixtures. In *Neural Information Processing Systems (NIPS)*, Vancouver, B.C., 2006.

[14] G. McLachlan and D. Peel. *Finite Mixture Models*. John Wiley & Sons, 2000.

[15] Thomas Minka and Zoubin Ghahramani. Expectation propagation for infinite mixtures. In *Neural Information Processing Systems (NIPS)*, Vancouver, B.C., 2003.

[16] Daniel J. Navarro, Thomas L. Griffiths, Mark Steyvers, and Michael D. Lee. Modeling individual differences using Dirichlet processes. *Journal of Mathematical Psychology*, 50:101–122, 2006.

[17] Radford M. Neal. Markov chain sampling methods for Dirichlet process mixture models. *Journal of Computational and Graphical Statistics*, 9:249–265, 2000.

[18] M. Nykter, T. Aho, M. Ahdesmaki, P. Ruusuvuori, A. Lehmussola, and O. Yli-Harja. Simulation of microarray data with realistic characteristics. *BMC Bioinformatics*, 7:349, 2006.

[19] Carl Rasmussen. The infinite Gaussian mixture model. volume 12. MIT Press, 2000.

[20] Jayaram Sethuraman. A constructive definition of Dirichlet priors. *Statistica Sinica*, 4:639–650, 1994.

[21] Yee Whye Teh, Michael I. Jordan, Matthew J. Beal, and David M. Blei. Hierarchical Dirichlet processes. *Journal of the American Statistical Association*, 2006.
