# Supplementary material for: Automated Discovery of Functional Generality of Human Gene Expression Programs
Source: PLoS Comput Biol. 2007 Aug 10;3(8):e148. doi: 10.1371/journal.pcbi.0030148 (PMC1941755; doi:10.1371/journal.pcbi.0030148)
Supplement: Supplementary file 2 [file pcbi.0030148.st001.pdf]

**Ref.**	**Infectious agents or immunomodulatory molecules**	**Host cell-type(s)**	**No. time-series**	**No. time-points per series**
Nau 2002 [1]	Live pathogenic bacteria and bacterial cell components; representative Gram-positive, Gram-negative and Mycobacterial organisms	Primary macrophages	23	4-5 (24 hrs)
Nau 2003 [2]	Interferons and interleukins	Primary macrophages	5	5 (24 hrs)
Huang 2001 [3]	Representative pathogenic bacteria, fungi, and viruses and relevant components	Primary dendritic cells	9	7-8 (24-36 hrs)
Boldrick 2002 [4]	Live and heat-killed pathogenic bacteria avirulent strains and bacterial components	Primary peripheral blood mononuclear cells (PBMCs) and cell-culture macrophages	15	5-7 (6-24 hrs)
Pathan 2004 [5]	Live pathogenic bacteria	Whole blood cells	4	5 (24 hrs)
Guillemin 2002 [6]	Live pathogenic bacteria and mutant strains	Cell-culture gastric epithelial cells	6	5 (24 hrs)

Table S1: Summary of infection gene expression time-series data sets analyzed. All host cells in the experiments were human primary or cell-culture derived.

Nau *et al.* exposed primary human macrophages collected from different donors to a variety of live bacteria and bacterial cell components [1]. The macrophages were exposed to the infectious agent or component for twenty-four hours, and four to five time-points were collected. Organisms included representative species from the major classes of human bacterial pathogens: Gram-positive organisms (*Staphylococcus aureus* and *Listeria monocytogenes*), Gram-negative organisms (*Escherichia coli*, enterohemorrhagic *E. coli* O157:H7 [EHEC], *Salmonella typhi* and *Salmonella typhimurium*), and Mycobacteria (*Mycobacterium tuberculosis* and *Mycobacterium bovis*). The macrophages were also exposed to bacterial cell components, including those specific to Gram-negative organisms (lipopolysaccharide [LPS]) or Gram-positive organisms (lipoteichoic acid [LTA] and protein A), and components common to all three classes of bacteria (heat shock proteins [hsp65 and hsp70], muramyl dipeptide [MDP], formyl-methionine-leucine-phenylalanine [f-MLP] and mannosylated proteins [D-(+)-mannose]).

In a follow-up study, Nau *et al.* exposed human macrophages to five different immune-modulating molecules [2]. The molecules used were: interferon-alpha (IFN-α), interferon-beta (IFN-β), interferon-gamma (IFN-γ), interleukin 10 (IL-10) and interleukin 12 (IL-12). The macrophages were exposed for twenty-four hours and five time points were collected.

Huang *et al.* exposed primary human dendritic cells collected from different donors to several live infectious agents and relevant components of the agents [3]. Dendritic cells, which reside in tissues in an immature state, are involved in initiating both innate and adaptive immune responses. They recognize and phagocytose antigens, which then leads to their maturation, expression of co-stimulatory molecules, and subsequent interactions

with naive T-cells. Dendritic cells were exposed to representative organisms and relevant components: bacteria (*E. coli* and LPS), fungi (*Candida albicans* and mannan, a fungal cell-wall component) and viruses (Influenza A and synthetic dsRNA). Cells were exposed for twenty-four or thirty-six hours, and seven to eight time-points were collected.

Boldrick *et al.* exposed primary human peripheral blood mononuclear cells (PBMCs) from different donors and cell-line derived macrophages to several heat-killed and live bacterial pathogens, a bacterial component, avirulent bacterial strains, and immunostimulatory chemicals [4]. Cells were exposed to heat-killed *E. coli*, *S. aureus* and *Bordatella pertussis* bacteria for six, twelve, or twenty-four hours and five to seven time-points were collected. Additional experiments were done using live virulent *B. pertussis*, avirulent strains, and LPS from the virulent organism. Additionally, cells were exposed to ionomycin and phorbol 12-myristate 13-acetate (PMA), chemicals that induce cellular responses mimicking antigen exposure. The authors noted that heat-killed strains were used to reduce confounding effects from differential bacterial growth rates and cytotoxic effects on host cells. *B. pertussis* was chosen for live infections, because this organism is relatively slow-growing and is not known to cause significant cytotoxicity. The authors additionally noted that the use of peripheral blood mononuclear cells, which consist of diverse cell populations involved in both innate and adaptive immunity, had advantages because interactions among different immune cells might be observed.

Pathan *et al.* exposed whole human blood cells from two donors to the live pathogenic bacteria Neisseria meningitides [5]. Cells were exposed for twenty-four hours and five time-points were collected.

Guillemin *et al.* exposed cell-culture derived human gastric epithelial cells to the live pathogen *Helicobacter pylori*, a leading cause of peptic ulcers [6]. Cells were exposed for twenty-four hours and five time-points were collected. Guillemin *et al.* also exposed cells to four different *H. pylori* mutants deficient in various genes of the *cag* pathogenicity island, a contiguous collection of genes that confer virulence properties. Unlike the host cells used in the other data sets described above, gastric epithelial cells are not involved in principle immune system functions.

1. Nau GJ, Richmond JF, Schlesinger A, Jennings EG, Lander ES, et al. (2002) Human macrophage activation programs induced by bacterial pathogens. Proc Natl Acad Sci U S A 99: 1503-1508.
2. Nau GJ, Schlesinger A, Richmond JF, Young RA (2003) Cumulative Toll-like receptor activation in human macrophages treated with whole bacteria. J Immunol 170: 5203-5209.
3. Huang Q, Liu D, Majewski P, Schulte LC, Korn JM, et al. (2001) The plasticity of dendritic cell responses to pathogens and their components. Science 294: 870-875.
4. Boldrick JC, Alizadeh AA, Diehn M, Dudoit S, Liu CL, et al. (2002) Stereotyped and specific gene expression programs in human innate immune responses to bacteria. Proc Natl Acad Sci U S A 99: 972-977.

5. Pathan N, Hemingway CA, Alizadeh AA, Stephens AC, Boldrick JC, et al. (2004) Role of interleukin 6 in myocardial dysfunction of meningococcal septic shock. Lancet 363: 203-209.
6. Guillemin K, Salama NR, Tompkins LS, Falkow S (2002) Cag pathogenicity island-specific responses of gastric epithelial cells to Helicobacter pylori infection. Proc Natl Acad Sci U S A 99: 15136-15141.
