# Supplementary material for: Automated Discovery of Functional Generality of Human Gene Expression Programs
Source: PLoS Comput Biol. 2007 Aug 10;3(8):e148. doi: 10.1371/journal.pcbi.0030148 (PMC1941755; doi:10.1371/journal.pcbi.0030148)
Supplement: Supplementary file 3 [file pcbi.0030148.st004.pdf]

Table S4: Summary of selected Gene Ontology (GO) biological process categories or Kyoto Encyclopedia of Genes and Genomes (KEGG) pathways for which expression programs discovered in the infection time-series data are significantly enriched. As expected, many significant GO categories and KEGG pathways were specifically involved with response to infection. Interestingly, a substantial number of significant categories or pathways corresponded to signaling cascades. Further, there were also a number of significantly enriched biological processes or pathways not directly labeled as being infection-related, but that are involved with alterations in cellular physiology consistent with infection. Finally, there were some unexpected significant categories or pathways.

**Infection response**	**Signaling pathways**	**Other**
- Inflammatory response (GO:0006954)<br>- Response to virus (GO:0009615)<br>- Chemotaxis (GO:0006935)<br>- Positive regulation of T-cell proliferation (GO:0042102)<br>- Endogenous antigen processing via MHC class II (GO:0019886)<br>- Cytokine-cytokine receptor interaction (KEGG:hsa04060)<br>- Natural killer cell mediated cytotoxicity (KEGG:hsa04650)<br>- Leukocyte transendothelial migration (KEGG:hsa04670)<br>- Complement and coagulation cascades (KEGG:hsa04610)	- MAPKKK (GO:0000165 and KEGG:hsa04010)<br>- JAK-STAT (GO:0007259 and KEGG:hsa04630)<br>- Toll-like receptor (KEGG:hsa04620)<br>- B-cell receptor (KEGG:hsa04662)<br>- T-cell receptor (KEGG:hsa04660)<br>- Insulin (KEGG:hsa04910)<br>- VEGF (KEGG:hsa04370)<br>- Calcium (KEGG:hsa04020)<br>- Wnt (KEGG:hsa04310)<br>- Phosphatidylinositol (KEGG:hsa04070)<br>- I-κB kinase/NF-κB (GO:0043123)<br>- Transmembrane receptor protein tyrosine kinase (GO:0007169)	- Apoptosis (GO:0006915)<br>- Nucleotide-excision repair (GO:0006289)<br>- Nuclear mRNA splicing (GO:0000398)<br>- Glycolysis (GO:0006096)<br>- Glycogen metabolism (GO:0005977)<br>- Anti-apoptosis (GO:0006916)<br>- Cell cycle (GO:00070490)<br>- Positive regulation of cell proliferation (GO:0008284)<br>- Tricarboxylic acid cycle (GO:000609)<br>- Regulation of adenylate cyclase activity (GO:0045761)<br>- Chloride transport (GO:0006821)<br>- Focal adhesion (KEGG:hsa04510)<br>- Oxidative phosphorylation<br>- (KEGG:hsa00190)<br>- Proteasome (KEGG:hsa03050)<br>- Androgen and estrogen metabolism (KEGG:hsa00150)<br>- Regulation of actin cytoskeleton (KEGG:hsa04810)<br>- Gap junction (KEGG:hsa04540)<br>- Fatty acid metabolism (KEGG:hsa00071)<br>- Ribosome (KEGG:hsa03010)<br>- Neuroactive ligand-receptor interaction (KEGG:hsa04080)
