# Supplementary material for: Automated Discovery of Functional Generality of Human Gene Expression Programs
Source: PLoS Comput Biol. 2007 Aug 10;3(8):e148. doi: 10.1371/journal.pcbi.0030148 (PMC1941755; doi:10.1371/journal.pcbi.0030148)
Supplement: Supplementary file 4 [file pcbi.0030148.st006.pdf]

Table S6: Manual classification of Novartis Gene Atlas v2 tissue samples into 10 high-level, physiologically based categories.

**Category**	**Tissues**
Neural	h_Amygdala, h_DRG, h_Hypothalamus, h_OlfactoryBulb, h_TrigeminalGanglion, h_cerebellum, h_spinalcord, h_TemporalLobe, h_globuspallidus, h_CerebellumPeduncles, h_caudatenucleus, h_WholeBrain, h_ParietalLobe, h_MedullaOblongata, h_PrefrontalCortex, h_OccipitalLobe, h_Thalamus, h_subthalamicnucleus, h_CingulateCortex, h_Pons, h_fetalbrain, h_ciliaryganglion, h_atrioventricularnode, h_SuperiorCervicalGanglion, m_amygdala, m_dorsal root ganglia, m_hypothalamus, m_olfactory bulb, m_trigeminal, m_cerebellum, m_spinal cord lower, m_substantia nigra, m_spinal cord upper, m_preoptic, m_frontal cortex, m_cerebral cortex, m_dorsal striatum, m_hippocampus, m_eye, m_medial olfactory epithelium, m_vomeralnasal organ
Muscle	h_Heart, h_SkeletalMuscle, h_TONGUE, h_CardiacMyocytes, h_SmoothMuscle, m_heart, m_skeletal muscle, m_tongue
Hematological/Immune	h_PB-CD4+Tcells, h_PB-CD8+Tcells, h_bonemarrow, h_lymphnode, h_thymus, h_WHOLEBLOOD, h_BM-CD33+Myeloid, h_PB-CD14+Monocytes, h_PB-BDCA4+Dentritic_Cells, h_PB-CD56+NKCells, h_PB-CD19+Bcells, h_BM-CD105+Endothelial, h_BM-CD34+, h_Tonsil, h_BM-CD71+EarlyErythroid, m_CD4+T-cells, m_CD8+T-cells, m_bone marrow, m_lymph node, m_thymus, m_bone, m_spleen, m_B220+ B-cells
Malignant	h_ColorectalAdenocarcinoma, h_leukemialymphoblastic(molt4), h_721_B_lymphoblasts, h_lymphomaburkittsRaji, h_leukemiapromyelocytic(hl60), h_lymphomaburkittsDaudi, h_leukemiachronicmyelogenous(k562)
Respiratory	h_Lung, h_fetallung, h_bronchialepithelialcells, m_lung, h_trachea, m_trachea
Renal	h_kidney, m_kidney, m_bladder
Endocrine	h_Thyroid, h_adrenalgland, h_fetalThyroid, h_PancreaticIslets, h_AdrenalCortex, m_pituitary, m_thyroid, m_adrenal gland, h_Pituitary
Reproductive/embryological	h_Ovary, h_PLACENTA, h_Uterus, h_testis, h_TestisLeydigCell, h_TestisGermCell, h_TestisInterstitial, h_TestisSeminiferousTubule, h_UterusCorpus, m_ovary, m_placenta, m_uterus, m_testis, m_embryo day 10.5, m_embryo day 9.5, m_embryo day 8.5, m_embryo day 7.5, m_embryo day 6.5, m_umbilical cord, m_mammary gland (lact), m_blastocysts, m_fertilized egg, m_oocyte, h_Prostate, m_prostate
Digestion/nutrient processing and storage	h_Liver, h_Pancreas, h_salivarygland, h_fetalliver, h_Appendix, m_liver, m_pancreas, m_salivary gland, m_stomach, m_large intestine, m_small intestine, h_ADIPOCYTE, m_adipose tissue, m_brown fat
Epidermis	h_skin, m_epidermis, m_digits, m_snout epidermis
