# Supplementary material for: Automated Discovery of Functional Generality of Human Gene Expression Programs
Source: PLoS Comput Biol. 2007 Aug 10;3(8):e148. doi: 10.1371/journal.pcbi.0030148 (PMC1941755; doi:10.1371/journal.pcbi.0030148)
Supplement: Supplementary file 5 [file pcbi.0030148.st007.pdf]

Table S7: Manual classification of tissue samples from the Shyamsundar *et al*. data set into 8 high-level, physiologically based categories.

Category	Tissues
Neural	Brain, frontal cortex 2271; Brain, frontal cortex 2273; Brain, frontal cortex 2272; Brain, temporal cortex 2273; Brain, temporal cortex 2272; Brain, occipital cortex 2271; Brain, occipital cortex 2273; Brain, occipital cortex 2272
Muscle	Heart 0024; Heart 0559; Heart 0477; Heart 0841; Heart 0980; Heart 2869; Muscle, abdominal 0031; Muscle, right calf 0315; Diaphragm 0366; Pericardium 0465
Hematological/Immune	Lymph node 2096; Lymph node, axillary 1187; Lymph node, axillary 0936; Lymph node 0599; Lymph node 1337; Tonsil 2852; Tonsil 3011; Tonsil 1428; Tonsil 1398; Thymus 0035; Thymus 0512; Spleen 0125; Spleen 0405; Spleen 0089; Buffycoat 3642; Buffycoat 3643
Respiratory	Lung 1356; Lung 1351; Lung 0221; Lung 0330
Renal	Kidney 0265; Kidney 0088; Kidney 1651; Kidney 0226; Kidney 1594; Bladder 1004; Bladder 1678
Endocrine	Thyroid 0029; Thyroid 0555; Thyroid 3077; Thyroid 0182; Thyroid 0838; Thyroid 1193; Parathyroid 2995; Parathyroid 0499 Parathyroid 1748; Adrenal 0558; Adrenal 0433; Adrenal 1354; Adrenal 1111
Reproductive	Prostate 0782; Prostate 0805; Prostate 0845; Prostate 1277; Prostate 1045; Seminal vesicle 0235; Seminal vesicle 0233; Seminal vesicle 0234; Epididymus 2125; Testes 0553; Testes 1068; Testes 1853; Ovary 0408; Ovary 0466; Ovary 1080; Ovary 0538; Ovary 0314; Fallopian tube 065A; Fallopian tube 065B; Fallopian tube 2386; Fallopian tube 2184; Uterus, endomyometrium 0126 Uterus, endomyometrium 002B; Uterus, endomyometrium 0002; Uterus, endomyometrium 0158; Uterine corpus, myometriun 1205; Cervix, endo cervical canal 1200; Cervix 2385; Cervix 2209; Vagina 0304; Placenta 2876; Breast, lactating 0162
Digestion/nutrient processing and storage	Salivary gland, parotid 0493; Salivary gland, parotid 1762; Salivary gland, parotid 0396; Salivary gland, parotid 0506; Esophagus 0331; Esophagus 0022; Esophagus 0406; Stomach, body 0328; Stomach, body 0468; Stomach, fundus 0878; Stomach, pylorus 2173; Small bowel, duodenum 0825; Small bowel, duodenum 2174; Small bowel, ileum 0359; Colon, ascending 0222; Colon 2075; Colon, sigmoid 0361; Pancreas 0432; Pancreas 2650; Liver 0560; Liver 0586; Liver 0032; Liver 1267; Liver 1274; Gallbladder 2131
